# Development and validation of a promising 5-gene prognostic model for pediatric acute myeloid leukemia

**DOI:** 10.1186/s43556-023-00162-y

**Published:** 2024-01-02

**Authors:** Yu Tao, Li Wei, Norio Shiba, Daisuke Tomizawa, Yasuhide Hayashi, Seishi Ogawa, Li Chen, Hua You

**Affiliations:** 1 Laboratory for Excellence in Systems Biomedicine of Pediatric Oncology, Department of Pediatric Hematology and Oncology, Chongqing Key Laboratory of Pediatrics, Ministry of Education Key Laboratory of Child Development and Disorders, China International Science and Technology Cooperation Base of Child Development and Critical Disorders, National Clinical Research Center for Child Health and Disorders, Children’s Hospital of Chongqing Medical University, Chongqing, China; 2 NHC Key Laboratory of Birth Defects and Reproductive Health, Chongqing Population and Family Planning Science and Technology Research Institute, Chongqing, China; 3 Chongqing Hospital of Traditional Chinese Medicine, Chongqing, China; 4 Department of Pediatrics, Yokohama City University Graduate School of Medicine, Yokohama, Japan; 5 Division of Leukemia and Lymphoma, Children’s Cancer Center, National Center for Child Health and Development, Tokyo, Japan; 6 Department of Hematology/Oncology, Gunma and Institute of Physiology and Medicine, Gunma Children’s Medical Center, Jobu University, Gunma, Japan; 7 Department of Pathology and Tumor Biology, Kyoto University, Kyoto, Japan; 8 Institute for the Advanced Study of Human Biology (WPI–ASHBi), Kyoto University, Kyoto, Japan; 9 Department of Medicine, Center for Hematology and Regenerative Medicine, Karolinska Institute, 17177 Stockholm, Sweden; 10 Department of Cellular and Genetic Medicine, School of Basic Medical Sciences, Fudan University, Shanghai, 200032 China

**Keywords:** Pediatric acute myeloid leukemia, Risk stratification, Prognostic, C-index

## Abstract

Risk classification in pediatric acute myeloid leukemia (P-AML) is crucial for personalizing treatments. Thus, we aimed to establish a risk-stratification tool for P-AML patients and eventually guide individual treatment. A total of 256 P-AML patients with accredited mRNA-seq data from the TARGET database were divided into training and internal validation datasets. A gene-expression-based prognostic score was constructed for overall survival (OS), by using univariate Cox analysis, LASSO regression analysis, Kaplan–Meier (K-M) survival, and multivariate Cox analysis. A P-AML-5G prognostic score bioinformatically derived from expression levels of *5* genes (*ZNF775*, *RNFT1*, *CRNDE*, *COL23A1*, and *TTC38*), clustered P-AML patients in training dataset into high-risk group (above optimal cut-off) with shorter OS, and low-risk group (below optimal cut-off) with longer OS (*p* < 0.0001). Meanwhile, similar results were obtained in internal validation dataset (*p* = 0.005), combination dataset (*p* < 0.001), two treatment sub-groups (*p* < 0.05), intermediate-risk group defined with the Children's Oncology Group (COG) (*p* < 0.05) and an external Japanese P-AML dataset (*p* = 0.005). The model was further validated in the COG study AAML1031(*p* = 0.001), and based on transcriptomic analysis of 943 pediatric patients and 70 normal bone marrow samples from this dataset, two genes in the model demonstrated significant differential expression between the groups [all log2(foldchange) > 3, *p* < *0.001*]. Independent of other prognostic factors, the P-AML-5G groups presented the highest concordance-index values in training dataset, chemo-therapy only treatment subgroups of the training and internal validation datasets, and whole genome-sequencing subgroup of the combined dataset, outperforming two Children's Oncology Group (COG) risk stratification systems, 2022 European LeukemiaNet (ELN) risk classification tool and two leukemic stem cell expression-based models. The 5-gene prognostic model generated by a single assay can further refine the current COG risk stratification system that relies on numerous tests and may have the potential for the risk judgment and identification of the high-risk pediatric AML patients receiving chemo-therapy only treatment.

**Supplementary Information:**

The online version contains supplementary material available at 10.1186/s43556-023-00162-y.

## Introduction

Pediatric acute myeloid leukemia (P-AML) accounts for 15–20% of all childhood acute leukemia, which could be classified based on morphology, lineage, and genetics [1]. Over the past three decades, the overall survival (OS) rates of children with AML have increased dramatically, with the present 5-year survival rate varying between 65 and 75%, and the initial complete remission rates around 80% after the induction chemotherapy [2–6]. However, it remains a catastrophic disease with around 40% relapsed patients [6] and efforts to develop novel target therapies and cell therapies to enhance OS in these patients are ongoing.

The prognosis of P-AML is determined by a variety of cytogenetic and molecular traits [7]. Clinical protocol design has placed a strong emphasis on risk stratification of therapy for P-AML in order to maximize treatment for high-risk groups while minimizing therapeutic intensity for lower-risk groups. According to the groups participating in the pediatric cooperative clinical trials, different risk variables are employed for stratification [8]. Historically, risk-groups and treatments were categorized in the Children's Oncology Group (COG) AAML0531 research based on the baseline genetic prognostic indicators and the disease responses following induction therapy [9]. The three-year event-free survival (EFS) in the low-risk, intermediate-risk, and high-risk groups of AAML0531 COG classification was 64.0%, 46%, and 27%, respectively, resulting in substantial survival disparities across the groups. However, 60% of pediatric AML cases lack chromosomal abnormalities that stratify prognosis, while 20% of them lack all recognized markers [10]. In addition, it is generally difficult to quantify how these mutational signatures interact to affect survival. Measurement of measurable residual disease (MRD) by multidimensional flow cytometry has allowed the categorization of individuals without genetic anomalies associated with treatment results, however, the accuracy of the MRD measurement is closely related to the selection of methodology and antigen [10].

With the advancement of new molecular biology technologies, risk-stratification systems have begun to incorporate elements from high-throughput sequencing, such as somatic mutation profiling discovered by genome sequencing and gene expression profiling based on microarray or RNA sequencing [11–15]. One of the most recent risk categorization methods for pediatric AML has been used in the ongoing COG phase III study AAML1831 [8], and the high-risk group was further expanded with 6 alterations by using the whole-genome sequencing data from two sequential COG phase III trials (NCT01407757 and NCT01371981) for de novo pediatric AML patients to interrogate structural and molecular alternations in the associated genes [16]. As Adam J. Lamble et al. reported, the number of patients assigned to the allogenic donor stem cell transplantation cohort could have been increased by this expanded COG risk assessment algorithm (expanded_COG_AAML1831) [16]. The 2017 European LeukemiaNet (ELN) risk stratification system combines cytogenetic abnormalities and genetic mutations to provide guidance on the risk stratification of AML patients and is routinely used in clinical practice for adult AML patients [17]. Major strides have been made in our understanding of the AML pathophysiology recently, including the identification of the molecular etiology of the disease. The updated 2022 ELN guideline presented better performance in stratifying survival between adult patients with intermediate- or high-risk AML treated with induction chemotherapy [18, 19]. However, the distinct molecular profiling of AML in pediatric and adult patients limits the application of the models established in adult cohort to the pediatric population [20].

While it is challenging to apply most of the above-mentioned models extensively in the clinical environment since some have too many genes for ease of assay performance, some have interactions between the incorporated molecular makers, and some have mediocre risk stratification effectiveness in P-AML, new molecular biomarkers are needed for better prognostic classification, ultimately, better therapeutic targets of P-AML [21]. In this study, we aimed to establish a prognostic score for P-AML based on gene expression profile from public databases and validate its stability and forecasting performance, which may provide guidance for the choice of therapy and follow-up in P-AML.

## Results

### Patient characteristics

**Fig. 1:**
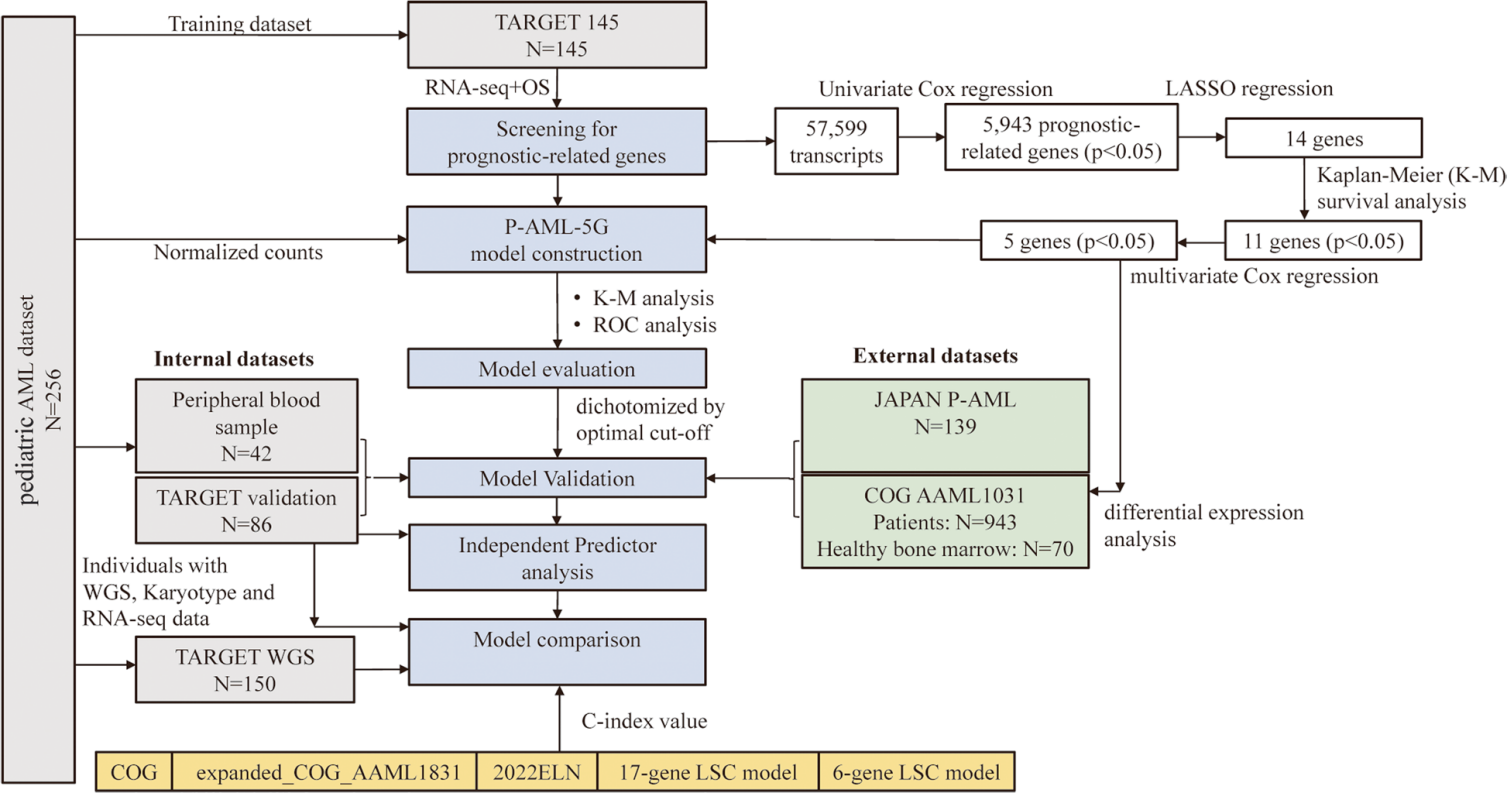
Overall design of this study

Clinical characteristics of the P-AML patients in TARGET 256, training dataset TARGET 145, validation datasets (TARGET 111, AAML1031 and JAPAN P-AML) can be found in Table S1. Based on the data from the cohorts mentioned above, we established and validated a prognostic model for pediatric AML. We also explored the potential clinical significance of the model and compared its prognostic ability with several existing models. Workflow of the study was presented in Fig. 1.

**Fig. 2:**
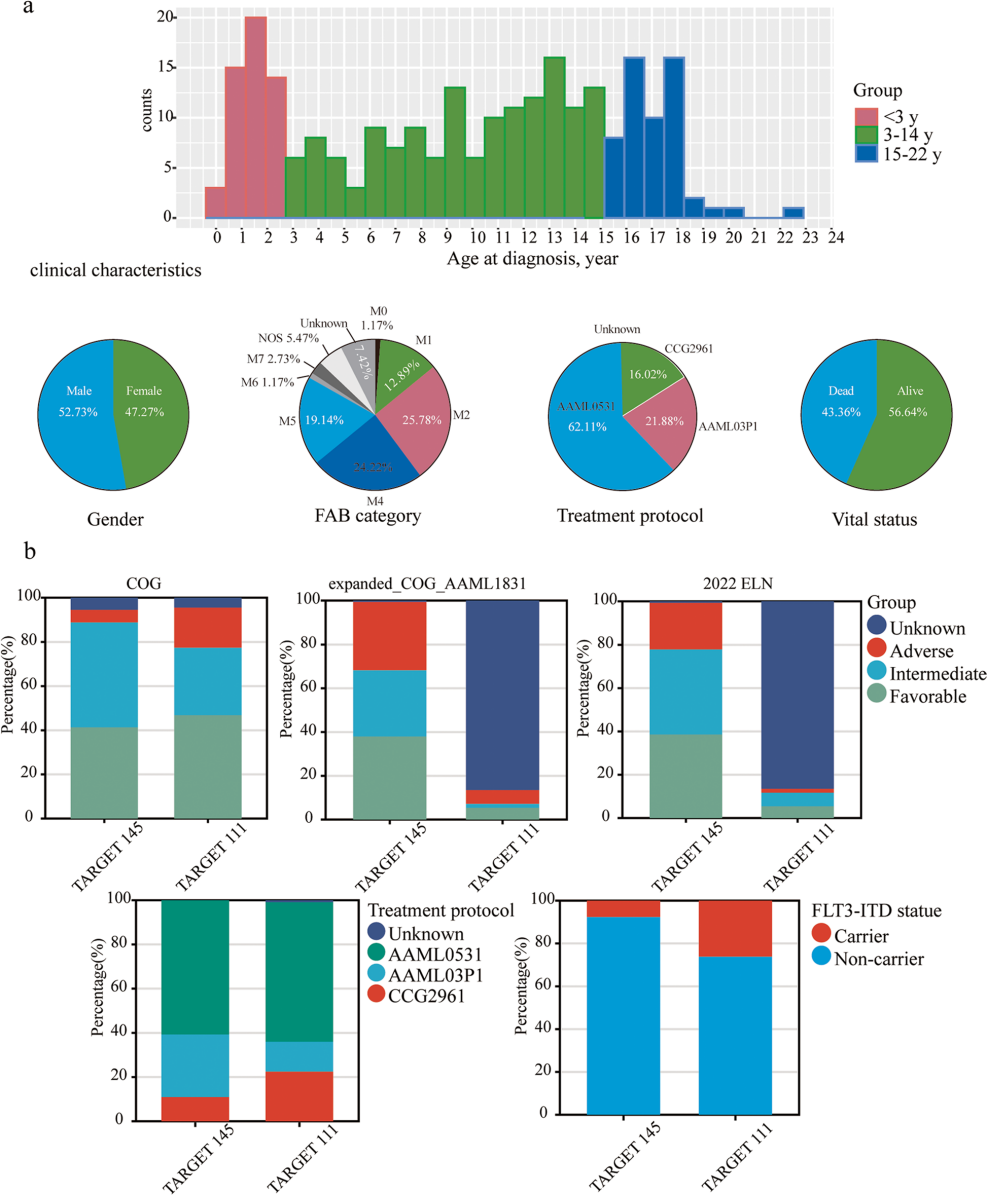
Clinical characteristics of the P-AML patients in TARGET 256. **a** The distribution of age at diagnosis (top panel), gender, FAB (Leukemia French American British Morphology Code), treatment protocol and vital status of pediatric AML patients in TARGET 256 (middle panel); **b** Clinical characteristics with significant differences between TARGET 145 and TARGET 111 (*p* < 0.01)

The distribution of age at diagnosis, gender, FAB (Leukemia French American British Morphology Code), treatment protocol and vital status of pediatric AML patients in TARGET 256 was shown in Fig. 2a and Table S1. The median age of all evaluated patients was 10.5 years (range, 10 days–22 years) and 135 patients (52.73%) were male. A total of 159 out of 256 patients (62.11%) received AAML0531 therapy, 56 (21.88%) received AAML03P1 therapy, and 41 (16.02%) received CCG-2961 therapy. The type or timing of induction therapy was randomized (CCG-2961 therapy) [22] or gemtuzumab ozogamicin (GO) was administered in a single-arm pilot (AAML03P1) [23] or a randomized fashion (AAML0531) [9]. Relapse as the first event occurred in 149 (58.20%) patients, and 47 (18.36%) patients had measurable residual disease (MRD1), defined as > 0.02% disease detected in the bone marrow by central difference from normal (ΔN) flow cytometry analysis after first course of induction chemotherapy [24, 25]. The percentage of hematopoietic stem cell transplantation (HSCT) was 12.50% (*N* = 32).

There were significant differences in COG, expanded_COG_AAML1831 and 2022ELN risk-group distribution, treatment protocol, GO treatment and FLT3_ITD (FLT3 Internal Tandem Duplication present) distribution between the TARGET 145 and TARGET 111 cohort (all *p* < 0.01, Fig. 2b). Compared to TARGET 145, more individuals received CCG2961 treatment protocol in TARGET 111 (22.52% vs. 11.03%, *p* < 0.01), more individuals were FLT3_ITD carriers (26.13% vs. 7.59%, *p* < 0.001), and less individuals received GO treatment (42.34% vs. 62.07%, *p* < 0.001). Considering the treatment subgroups in TARGET 256, P-AML patients received the AAML03P1 protocol presented the best prognosis (Fig. S1). These data revealed that TARGET 145 and TARGET 111 are two distinct datasets with clinical differences, akin to real-world scenarios of training and validation datasets.

### Construction of the prognostic risk score

**Fig. 3:**
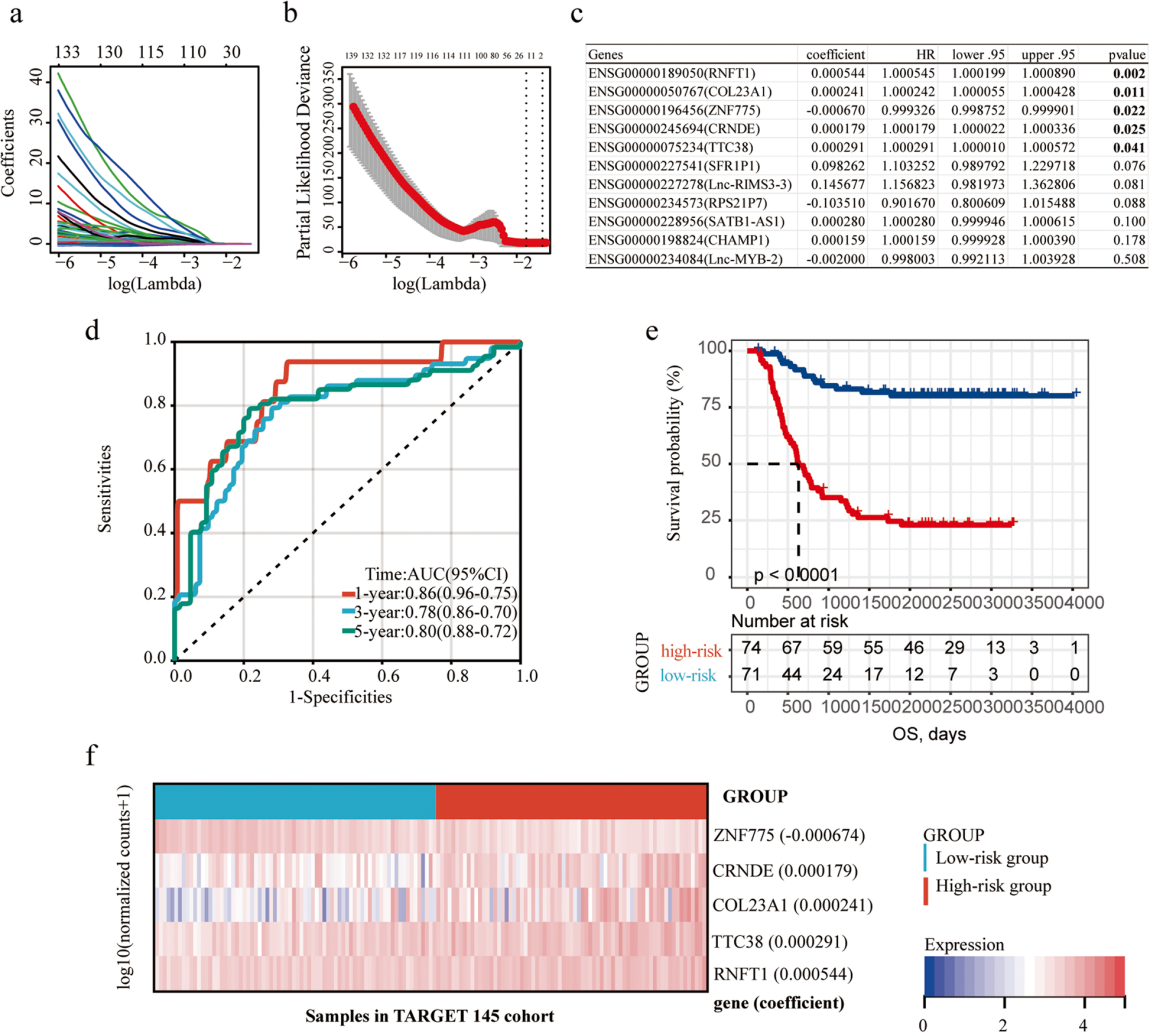
Prognostic model construction. **a** Each curve represented the changed trajectory of each prognosis-related gene variable coefficient. When fourteen variables remained, the lowest partial probability of deviance was observed; **b** A coefficient spectrum was generated for screening variables. The first dotted vertical lines at optimal log (Lambda) value; **c** A total of five genes were identified according to multivariate cox regression analysis to construct the prognostic model; **d** ROC analysis of gene signature for prediction of OS risk at 1, 3, and 5 years in TARGET 145 dataset; **e** Kaplan–Meier curves of OS based on prognosis model (*p* < 0.001); **f** Expression profiles of 5 genes in the high-risk group and the low-risk group. OS: overall survival; ROC: receiver operating characteristic

Our clinical hypothesis is that gene RNA expression levels may serve as reliable and convenient prognostic indicators. To investigate this issue, we conducted univariate Cox regression analysis of 57,599 genes in the TARGET 145 dataset. As a result, 5,943 genes were identified as the potential biomarkers for predicting the patient-specific OS (based on a threshold of *p* < 0.05 for individual gene analyses). Among these 5,943 genes, the LASSO-Cox regression analysis identified 14 genes that were the most relevant to OS prognosis (Fig. 3a b). Of these, expression levels of 11 genes (dichotomized by their median expression levels) were individually correlated with either a longer or a shorter OS according to the K-M survival curves (Fig. S2) and subsequently subjected to the multivariate Cox regression analysis. Notably, the results revealed that five genes were the independent predictors of OS (Fig. 3c, *p* < 0.05). A five-gene risk score (P-AML-5G) was then established by integrating the expression levels (normalized counts) and the coefficients derived from multivariable Cox regression analyses, and the formula was exhibited as below: Risk Value = [0.00024149 × *COL23A1* expression] + [0.00029096 × *TTC38* expression] + [0.00054436 × *RNFT1* expression]- [0.000674 × *ZNF775* expression] + [0.0001792 × *CRNDE* expression]. ROC curve analysis showed that area under the ROC curve (AUC)s of P-AML-5G score for 1-, 3- and 5-year OS were 0.86, 0.78, 0.80, respectively (Fig. 3d). The optimal cutoff risk value (1.676) was used further to divide patients into high- and low-risk groups. Patients in the low-risk group had a significantly longer OS than of those in the high-risk group (*p* < 0.001) (Fig. 3e). Patients in the low-risk group also had a significantly longer event-free survival (EFS) than of those in the high-risk group (all *p* < 0.001) (Fig. S3a). AUCs for 1-, 3- and 5-year EFS were 0.71, 0.70, 0.71, respectively (Fig. S3b). Heatmap showing the differential expression of five genes between the high- and low-risk groups (Fig. 3f). The results revealed that the expressions of *COL23A1*, *TTC38*, *RNFT1*, and *CRNDE* genes were significantly increased in the high-risk group compared with the low-risk group, while the expression of *ZNF775* was significantly reduced in the high-risk group (Fig. S4).

### Prognostic value validation

**Fig. 4:**
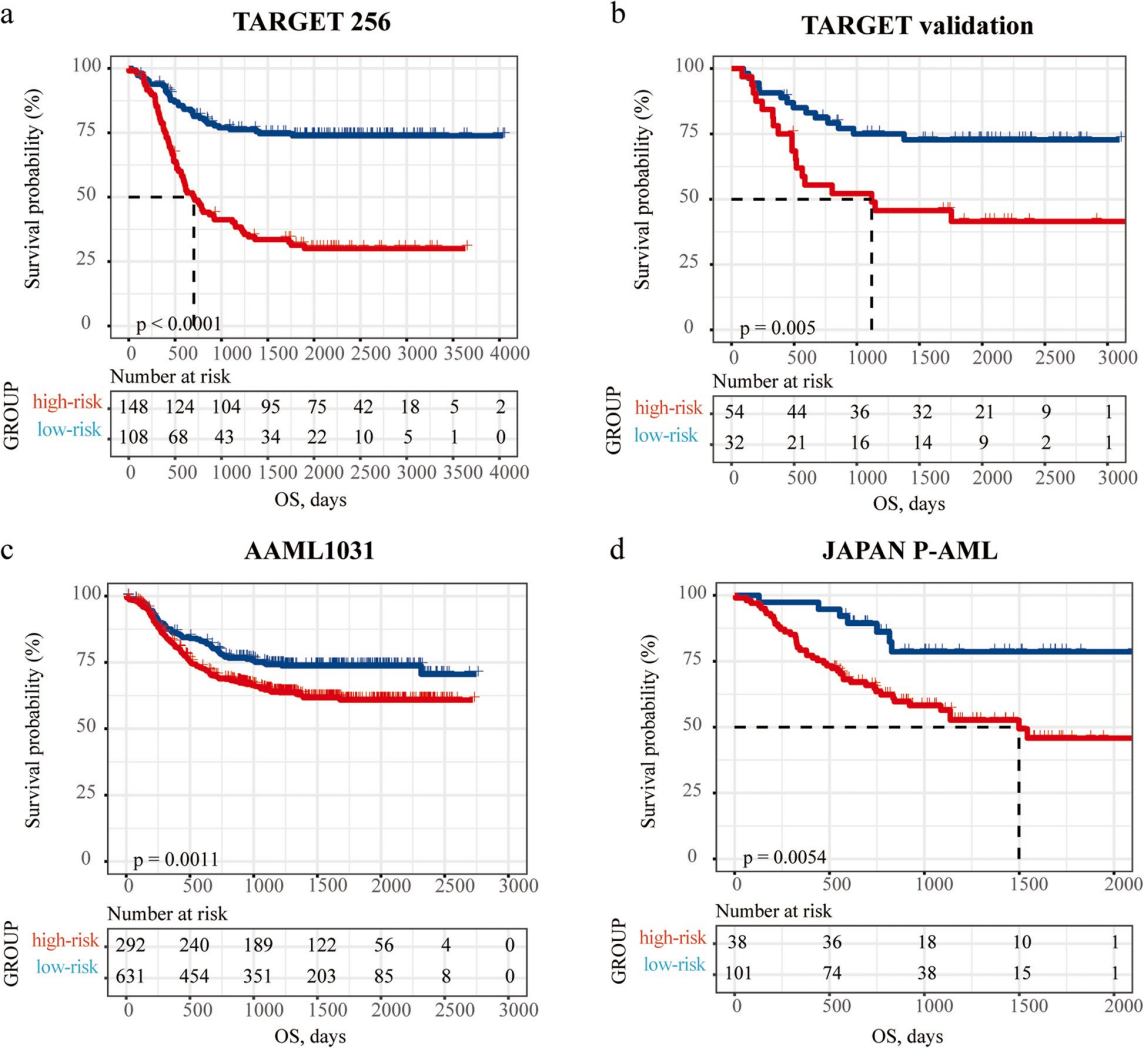
Prognosis model validation in internal and external datasets. In **a** TARGET 256, **b** TARGET validation, **c** AAML1031 and, **d** JAPAN P-AML, patients with high-risk scores had worse OS than patients with low-risk scores (all *p* < 0.01)

Further model validation results are essential to demonstrate the stability and feasibility of the model. As expected, patients in the high-risk group presented significantly shorter OS than those of the patients in the low-risk group in TARGET 256 (*p* < *0.001; HR* = *3.74, 95CI% (2.52, 5.57*) (Fig. 4a). Considering the treatment subgroups in the TRGET 145 dataset, the P-AML-5G risk groups provided powerful stratified performance in patients received AAML03P1 therapy (*p* < 0.001; Fig. S5a) and AAML0531 therapy (*p* < 0.001; Fig. S5b), while the result was unsatisfactory for stratifying patients received CCG2961 therapy (*p* = 0.31; Fig. S5c). Therefore, patients receiving protocol AAML0531 and AAML03P1 in TARGET 111 dataset were kept for internal model validation (N = 86, hereafter TARGET validation). The results showed that OS and EFS of the patients in the high-risk group were significantly shorter than those of the patients in the low-risk group (*p* = *0.005; HR* = *2.63, 95CI% [1.30, 5.26]*; *p* = *0.000; HR* = *3.10, 95CI% [1.73, 5.56]* (Fig. 4b and Fig. S6). We further demonstrated its prognostic generality in AAML1031 and JAPAN P-AML datasets with univariate Cox regression analysis [HR = 1.377, 95%CI (1.117 ~ 1.697), *p* = 0.003; HR = 1.756, 95%CI (1.103 ~ 2.793), *p* = 0.018, respectively]. A notable survival contrast was observed between the high and low-risk groups, as determined by the optimal split point identified in both of these datasets [*p* = *0.001; HR* = *1.55, 95CI% (1.19, 2.01*); *p* = *0.005; HR* = *2.94, 95CI% (1.33, 6.53*), respectively] (Fig. 4c-d). In comparison to the bone marrow tissue of healthy children, the RNA levels of *COL23A1* and *CRNDE* exhibited significant upregulation in the bone marrow of pediatric patients participating in the AAML1031 study [all log2(foldchange) > 3, *p* < 0.001] (Fig. S7). The aforementioned results revealed that our model exhibited prognostic capability in multiple pediatric AML validation datasets, and two genes in the model showed significant differential expression between patients and controls.

### Screening for prognostic factors for OS in the P-AML dataset

To investigate whether the P-AML-5G model is an independent prognostic factor for p-AML, we performed a univariate Cox regression analysis for screening the prognostic factors for OS using the P-AML-5G groups, COG, along with other clinical and/or genetic markers. Nine variables, including the P-AML-5G groups, treatment protocol, inv(16), MinusX, Cytogenetic Complexity, FLT3_ITD, WT1 mutation, COG risk stratification system and CBFB-MYH11 fusion were potential risk factors affecting OS in the TARGET 145 (*p* < 0.01, Table 1). Furthermore, multivariate survival analysis using the above variables found that the P-AML-5G groups was an independent prognostic factor for OS of the P-AML patients (*p* < 0.05, Table 2). In the TARGET validation dataset, the P-AML-5G groups, PB, COG risk stratification system and MRD1 were associated with the OS (*p* < 0.05) (Table 1), while P-AML-5G groups and COG risk system were independent prognostic factors (Table 2). Hence, the P-AML-5G groups offer independent prognostic information for the overall survival of pediatric AML across multiple datasets.

**Table 1:** Univariable Cox proportional hazards regression analysis of P-AML-5G

Datasets	TARGET 145				TARGET validation			
**Characteristic**	**N**	**HR**^a^	**95% CI**^a^	***p*-value**	**N**	**HR**^a^	**95% CI**^a^	***p*-value**
P-AML-5G groups	145				86			
Low-risk		Ref						
High-risk		6.646	3.671, 12.03	**0.000**		2.632	1.307, 5.301	**0.007**
Gender	145				86			
Female		Ref						
Male		0.680	0.421, 1.098	0.114		0.772	0.385, 1.545	0.464
Ethnicity	141				80			
Hispanic or Latino		Ref Ref						
Not Hispanic or Latino		0.644	0.361, 1.148	0.135	0.501	0.238, 1.055	0.069	0.501
Age at Diagnosis, days	145	1.000	1.000, 1.000	0.074	86	1.000	1.000, 1.000	0.575
Protocol	145				86			
AAML03P1		Ref						
AAML0531		1.883	1.014, 3.497	**0.045**		1.508	0.529, 4.301	0.442
CCG2961		3.367	1.507, 7.523	**0.003**		\	\	\
WBC at diagnosis,10^3^ mcl	145	0.999	0.996, 1.003	0.670	86	1.00	0.996, 1.003	0.785
BM at diagnosis, %	142	1.001	0.989, 1.013	0.859	82	0.987	0.970, 1.005	0.150
PM at diagnosis, %	145	0.997	0.988, 1.005	0.431	86	0.984	0.970, 0.997	**0.019**
CNS disease (yes vs. no)	145	1.561	0.675, 3.609	0.298	86	1.245	0.379, 4.088	0.718
Chloroma (yes vs. no)	144	0.694	0.253, 1.907	0.479	86	1.248	0.513, 3.035	0.625
c8_21 (yes vs. no)	135	0.871	0.414, 1.835	0.717	82	0.369	0.112, 1.218	0.102
inv(16) (yes vs. no)	135	0.417	0.189, 0.917	**0.030**	82	0.547	0.130, 2.296	0.409
t(9;11)(p22;q23) (yes vs. no)	134	0.841	0.336, 2.105	0.712	81	0.560	0.076, 4.118	0.569
del9q (yes vs. no)	135	1.072	0.262, 4.389	0.923	82	1.993	0.474, 8.382	0.347
trisomy8 (yes vs. no)	135	1.758	0.754, 4.099	0.191	82	1.078	0.327, 3.555	0.902
MLL (yes vs. no)	135	1.573	0.851, 2.907	0.149	82	1.353	0.518, 3.536	0.537
MinusY (yes vs. no)	135	0.750	0.183, 3.072	0.689	82	0.523	0.071, 3.841	0.524
MinusX (yes vs. no)	135	3.140	1.127, 8.745	**0.029**	82	0.000	0.000, Inf	0.998
Cytogenetic Complexity	138				82			
0 ~ 2		Ref						
3andmore		2.041	1.142, 3.650	**0.016**		0.725	0.220, 2.394	0.598
Primary Cytogenetic Code	135				82			
Normal		Ref						
Not-normal		1.155	0.601, 2.222	0.666		1.267	0.564, 2.847	0.567
FLT3_ITD (yes vs. no)	145	3.879	1.958, 7.685	**0.000**	86	1.298	0.614, 2.745	0.495
FLT3_PM (yes vs. no)	145	0.439	0.138, 1.398	0.164	84	0.868	0.207, 3.634	0.846
NPM (yes vs. no)	140	0.614	0.150, 2.512	0.498	84	0.565	0.172, 1.857	0.347
CEBPA (yes vs. no)	144	0.000	0.000, Inf	0.996	84	0.364	0.050, 2.664	0.319
WT1 (yes vs. no)	141	2.864	1.228, 6.680	**0.015**	84	1.199	0.286, 5.037	0.804
MRD1 (yes vs. no)	110	1.436	0.772, 2.670	0.253	72	2.351	1.078, 5.127	**0.032**
CR1	145				86			
CR		Ref						
not in CR		0.895	0.409, 1.958	0.781		1.462	0.629, 3.398	0.378
unevaluable		6.903	0.922, 51.66	0.060		7.849	1.002, 61.50	0.050
COG	137				83			
favorabl		Ref						
Intermed		2.622	1.464, 4.696	**0.001**		3.848	1.581, 9.368	**0.003**
adverse		5.845	2.387, 14.31	**0.000**		3.049	1.104, 8.416	**0.031**
HSCT (yes vs. no)	144	0.511	0.186, 1.404	0.193	83	1.427	0.635, 3.207	0.390
Gemtuzumab.ozogamicin.treatment (yes vs. no)	129	0.799	0.461, 1.387	0.425	86	0.604	0.301, 1.211	0.155
NUP98 fusion (yes vs. no)	112	1.893	0.924, 3.877	0.081	56	0.000	0.000, Inf	0.998
KMT2A-r (yes vs. no)	112	1.328	2.084, 2.336	0.323	56	1.131	0.411, 3.115	0.811
RUNX1-RUNX1T1 (yes vs. no)	112	0.797	0.402, 1.581	0.516	56	0.343	0.100, 1.172	0.088
CBFB-MYH11 (yes vs. no)	112	0.336	0.152, 0.743	**0.007**	56	0.486	0.113, 2.096	0.334

*WBC* The absolute peripheral white blood cell count, *BM* Bone Marrow Blast Cell Outcome Percentage Value, *PM* Peripheral Blast Cell Outcome Percentage Value, *Chloroma* Chloroma Disease At Diagnosis Present, *CNS* Central Nervous System Disease At Diagnosis Present, *c8_21* Chromosomal translocation between chromosome 8 and chromosome 21present, *t(9;11)(p22;q23* Cytogenetic Abnormality t(9;11)(p22;q23) present, *inv(16)* Cytogenetic Abnormality Chromosomal Inversion Chromosome 16 present, *del9q* Cytogenetic Abnormality Deletion Mutation 9q present, *trisomy8* Cytogenetic Abnormality Trisomy Chromosome 8 present, *MLL* Cytogenetic Abnormality Translocations Involving MLL1 (KMT2A) Gene present, *MinusY* Cytogenetic Abnormality Monosomy Chromosome Y Present, *MinusX* Cytogenetic Abnormality Monosomy Chromosome X Present, *Cytogenetic Complexity* Cytogenetic Abnormality Number of abnormalities, *Primary Cytogenetic Code* Cytogenetic Abnormality Predominant Classification Type, *FLT3_ITD* FLT3 Internal Tandem Duplication present, *NPM* mutation of the NPM gene present, *CEBPA* mutation of the CEBPA gene present, *WT1* mutation of the WT1 gene present, *FLT3_PM* FLT3 point mutation at codon 835–836 present, *MRD1* Minimal Residual Disease At End First Course, *CR1*: The remission status at the end of the first course of therapy determined by morphologic evaluation of marrow; < 5% blast, *CR; COG* Children's Oncology Group; Protocol: Children's Oncology Group Clinical Study Protocol, *HSCT* Stem Cell Transplant During First Complete Remission, *ELN* European LeukemiaNet, *NUP98 fusion* KMT2A-r, RUNX1-RUNX1T1, CBFB-MYH11: Gene Fusion by RNA or Whole Genome Sequencing or Karyotyping Identification

^a^*HR* Hazard Ratio, *CI* Confidence Interval

**Table 2:** Multivariable Cox Proportional Hazards Regression Analysis of P-AML-5G

Datasets	**TARGET 145**		
**Characteristic**	**HR**^a^	**95% CI**^a^	***p*-value**
P-AML-5G groups			
Low-risk	Ref		
High-risk	10.941	5.042, 23.743	**0.000**
MinusX			
No	Ref		
Yes	3.586	1.239, 10.379	**0.018**
FLT3_ITD			
No	Ref		
Yes	4.474	1.634, 12.252	**0.004**
	**TARGET validation**		
	**HR**^a^	**95% CI**^a^	***p*-value**
P-AML-5G groups			
Low-risk	Ref		
High-risk	2.482	1.103, 5.588	**0.028**
COG			
favorabl	Ref		
Intermed	5.454	1.771, 16.801	**0.003**
adverse	4.170	1.243, 13.989	**0.021**

*MinusX* Cytogenetic Abnormality Monosomy Chromosome X Present, *FLT3_ITD* FLT3 Internal Tandem Duplication present, *COG* Children's Oncology Group

^a^*HR* Hazard Ratio, *CI* Confidence Interval

### Clinical significance of the P-AML-5G model

Pre-treatment risk stratification, post-treatment MRD status, and identifying suitable patients for HSCT are critical indicators or events in the clinical management of p-AML. Therefore, we also investigated the correlation between P-AML-5G risk groups and these indicators to explore the potential clinical significance of P-AML-5G. According to the Sankey diagram of the P-AML-5G groups and COG risk groups, we found that P-AML-5G resulted in the reclassification of 100% (8/8) of COG adverse patients, 25.00% (15/60) of COG favorable and 65.20% (45/69) of COG intermediate patients in the high-risk group of the TARGET 145 dataset. In the TARGET validation dataset, P-AML-5G classified 30.00% (9/30) of COG favorable, 47.80% (11/23) of COG intermediate, and 43.80% (7/16) of COG adverse patients to the high-risk group (Fig. S8).

**Fig. 5:**
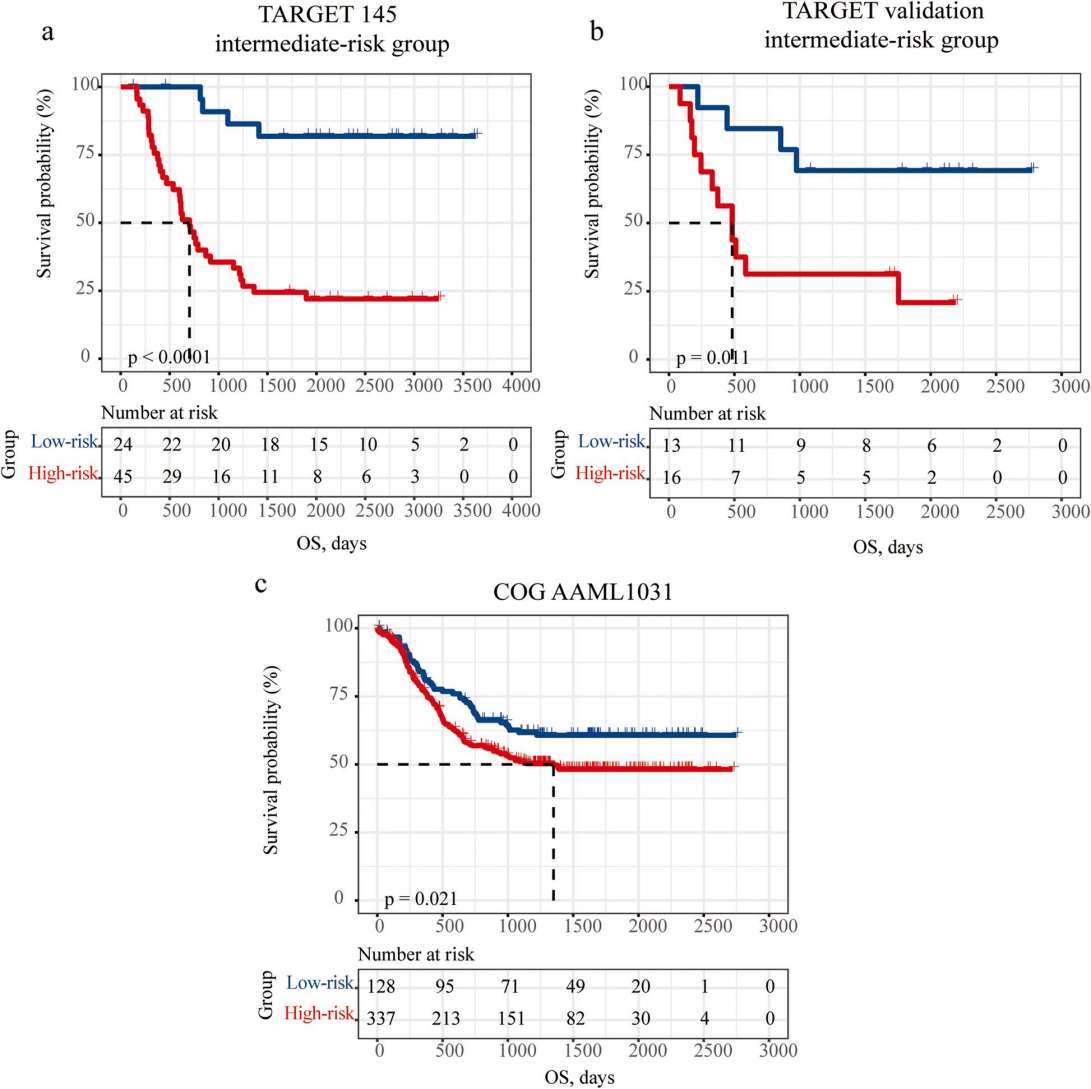
The P-AML-5G groups provided re-stratification value in the heterogeneous intermediate risk group defined by the traditional COG risk system in **a** TARGET 145, **b** TARGET validation and **c** AAML1031

Based on the above observations, we did Kaplan–Meier survival analysis for OS of the COG risk groups stratified by the P-AML-5G groups. We found that P-AML-5G groups provided prognostic information beyond that provided by COG. Noteworthy was that within intermediate risk groups, high-P-AML-5G score patient had worse prognosis than low-P-AML-5G score patients in TARGET 145, TARGET validation and AAML1031 (*p* < 0.001, *p* = 0.011*, p* = *0.021*; respectively, Fig. 5a-c).

Considering the missing rate of MRD1 and a low frequency of individuals receiving HSCT, the impact of the MRD1 and HSCT on the P-AML-5G risk groups was explored in the TARGET 256 dataset. MRD1 status was missing for 28.9% (74/256) of the patients. There were respectively 16.9% (25 of 148) and 20.4% (22/108) of subjects who were identified MRD1-positive in the low-, and high-risk categories. Although MRD1 had no impact on the P-AML-5G high-risk group (log-rank test, *p* = 0.22), MRD1-positive individuals in the P-AML-5G low-risk group presented a statistically suggestive worse prognoses compared to those with the low-risk/MRD1-negative patients (log-rank test, *p* = 0.09) (Fig. S9a). Including the four patients with incomplete HSCT information, there were 10.8% (16 of 148) and 14.8% (16/108) of individuals in the low-, and high-risk categories, respectively, who underwent HSCT. According to the K-M survival curves, HSCT showed the trend to improve the OS among the P-AML-5G high-risk patients (log-rank test, *p* = 0.20) (Fig. S9b).

In the TARGET 256 dataset, a total of 42 samples obtained from patients' peripheral blood were collected for further analysis. Our findings substantiate the model's significant capacity for risk stratification in relation to OS and the consistent trend observed for EFS in these peripheral blood samples (see Fig. S10) [*p* = *0.001; HR* = *4.76, 95CI% (1.67, 12.5*); *p* = *0.10; HR* = *1.85, 95CI% (0.870, 3.85*), respectively].

The above results demonstrated that using a single assay, the 5-gene prognostic model might enhance the current COG risk stratification system that currently relies on multiple tests, and has the potential to improve risk assessment for pediatric AML patients.

### Comparison with existing AML risk stratification tools

**Fig. 6:**
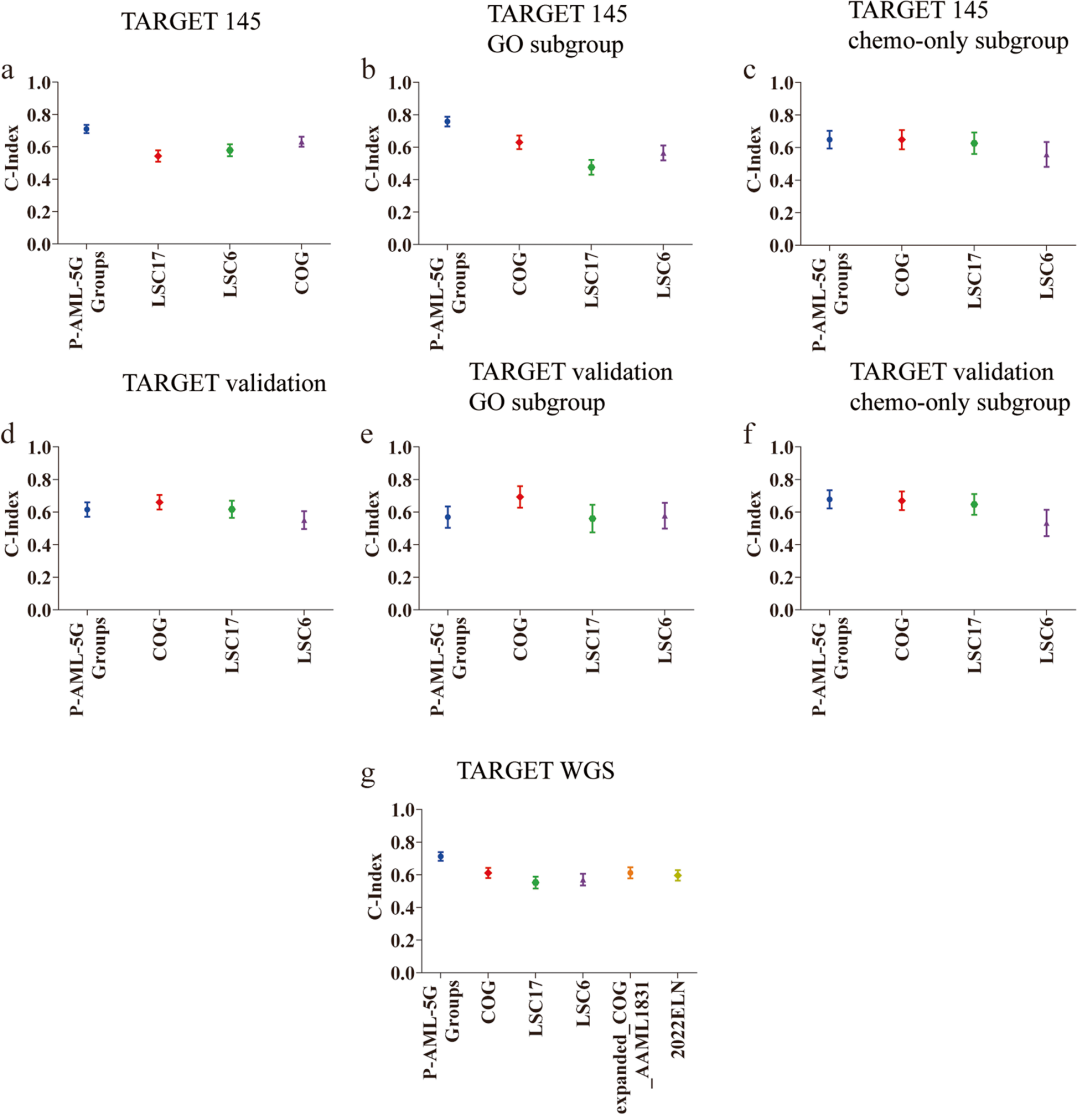
Model comparison of P-AML-5G with pre-existing AML risk classification tools. **a**, **c** Compared with pre-existing AML risk classification tools, the P-AML-5G groups had the highest C-index value in TARGET 145 and two treatment subgroups; **d**, **e** The COG risk system presented the highest C-index value in TARGET validation and GO treatment subgroup of the TARGET validation; **f** The P-AML-5G groups had the highest C-index value in chemo-therapy treatment only subgroup of TARGET validation dataset; **g** The P-AML-5G groups had the highest C-index value in the whole-genome sequenced subgroup

To demonstrate the clinical applicability of the P-AML-5G model, we compared its prognostic capability with previous models across multiple datasets. As shown in Table 3 and Fig. 6, compared to the COG, LSC17 model and LSC6 model, the P-AML-5G groups demonstrated the highest value of C-index in TARGET 145, GO treatment and chemotherapy-only treatment subgroups (0.71, 0.758, 0.648, respectively, Fig. 6a-c). In TARGET validation and GO treatment subgroup, the COG risk system presented the best C-index values (0.66 and 0.693) (Fig. 6d-e). However, subgroup analysis suggested that our P-AML-5G groups demonstrated the highest values of C-index in the chemotherapy-only treatment subgroups of both the TARGET 145 and TARGET validation dataset (0.648 and 0.678, Table 3, Fig. 6c, f). Our results revealed that existing clinical prognostic tools and published RNA-seq based models were outperformed by the P-AML-5G groups in the chemo-therapy subgroup of both the training and validation cohorts, which may have the potential for precision treatment in the pediatric AML.

**Table 3:** Comparison of P-AML-5G with pre-existing AML risk classification tools

	Concordance analysis for OS C-index (se)					
Datasets	P-AML-5G groups	COG	LSC17	LSC6	expanded_COG_AAML1831	2022ELN
**TAREGT 145**	**0.71(0.025)**	0.632(0.031)	0.543(0.035)	0.579(0.037)	\	\
GO	**0.758(0.03)**	0.63(0.042)	0.476(0.046)	0.564(0.046)	\	\
Chemo-only	**0.648(0.054)**	0.648(0.059)	0.626(0.066)	0.557(0.076)	\	\
**TARGET validation**	0.615(0.044)	0.660(0.045)	0.617(0.052)	0.551(0.055)	\	\
GO	0.569(0.066)	0.693(0.066)	0.560(0.085)	0.578(0.079)	\	\
Chemo-only	**0.678(0.056)**	0.669(0.057)	0.647(0.064)	0.533(0.081)	\	\
**TAREGT WGS**	**0.712(0.026)**	0.611(0.031)	0.552(0.036)	0.57(0.036)	0.612(0.034)	0.596(0.032)

*COG* Children's Oncology Group, *ELN* European LeukemiaNet, *LSC* leukemic stem cell, *OS* Overall Survival, *se* standard error

The exclusion of individuals with missing values for any of the three risk assignment tools (COG, 2022ELN, expanded_COG_AAML1831) in the combined dataset TARGET 256, left us with a total of 150 pediatric AML cases (named TARGET WGS hereafter). The P-AML-5G groups showed good model performance with the highest C-index value (0.712), outperformed COG, 2022ELN, expanded_COG_AAML1831, and two LSC models (Fig. 6g).

## Discussion

Prognostic guidance systems need to be adjusted as our knowledge of AML biology, cytogenetic and molecular characterizations, and the availability of novel treatment agents expand. This study established a cytogenic and genetic mutation independent five gene transcriptional signature, which may identify pediatric AML patients who will have negative outcomes at the time of diagnosis. The model has undergone successful validation in an internal validation set, as well as in two external validation sets. Notably, one of the external validation sets pertained to a Japanese population, characterized by a genetic background entirely distinct from that of the model's training cohort. In both the training and internal validation cohort, the risk model retained predictive value in multivariable analysis and improved risk prediction in the setting of traditional cytomolecular COG classification, especially in the chemo-therapy treatment-only subgroup.

A strong predictive biomarker of treatment outcomes would, in theory, early enough in the course of treatment identify a group of patients who had sufficiently high risk of recurrence and treatment resistance to warrant consideration of alternative therapies. In the training TARGET 145 and combined TARGET 256 datasets, the P-AML-5G risk groups in this study presented better performance in recognizing groups of high-risk patients from several clinical trials without the aid of predefined prognostic factors or COG risk system. Since the inclusion of the training dataset TARGET 145 in the combined cohort might unfairly bias the results towards the "trained" dataset, we further validate its independent prognostic value in the TARGET validation dataset. It should be highlighted that the TARGET dataset being enriched in poor performing AML and the training and validation sets exhibiting very different clinical characteristics. Meanwhile, our model also exhibits promising predictive capabilities for OS in a subset of peripheral blood samples, suggesting its clinical value in enabling risk assessment at an earlier stage with lower invasiveness. However, due to the limited involvement of only 42 peripheral blood samples in this study, further comprehensive investigations in larger population cohorts are warranted.

With respect to model comparison using C-index values, we would have expected the P-AML-5G groups to show the best model predictive power in the TARGET validation dataset, and then the results would have been very intriguing, considering the clinical convenience of RNA-seq assays versus cytological and mutation assays. This may be due to the small sample size of TARGET validation, differences in clinical characteristics from the training dataset and the fact that it includes people on two treatment regimens, which also reflects the high heterogeneity of p-AML patients at both the genomic and transcriptomic levels. However, the P-AML-5G is well-suited for future research and clinical use due to a number of properties. Firstly, P-AML-5G groups augments the existing standard COG risk system with new data, especially for re-stratifying heterogeneous populations now categorized as intermediate risk (over 40% of the combined cohort). Secondly, within patients receiving chemotherapy treatment only, P-AML-5G groups presented the best prognostic prediction performance. This result further suggests that a simple and straightforward gene expression assay panel, may have better performance in certain AML molecular subgroups or treatment subgroups. Thirdly, high-volume bone marrow is needed for many stages of testing according to the COG's current methodology, while gene-expression assay panel is much more economical, material and time saving, and facilitates the application in less developed areas. Based on above discussion, clinical use of the P-AML-5G model, either alone or in conjunction with traditional COG system, is completely consistent with the movement toward better risk stratification for P-AML.

With data from 150 whole genome-sequenced patients, we were for the first time able to compare P-AML-5G groups with several latest risk stratification systems for pediatric our adult AML. The ELN risk stratification system is universally accepted for the risk stratification of adult AML patients [26], however, its application in P-AML is not idealistic in previous studies [27, 28] and our analysis The fact that the ELN risk stratifications are unquantified and broad molecular differences between age cohorts may be to account for this result. Notably, we were unable to find a satisfactory prognostic value for the expanded_COG_AAML1831. The possible reasons might be the small sample size of the whole-genome sequenced individuals in this study or the complexity of the risk assignment for carriers with co-occurring mutations spanning risk categories (6 in 150 individuals within expanded_COG_AAML1831 groups) [29].

The significance of responsiveness to the first medication and evaluation of early MRD in the individual risk assignment is emphasized in addition to the baseline genetic characterization [24]. Although MRD1 did not hold independent prognostic implications for P-AML in this study, it was one of the prognostic indicators in the TARGET validation dataset. It was intriguing that the MRD1-positive patients in the low-risk P-AML-5G group had poorer trend of prognoses than that of the low-risk/MRD1-negative group patients. The strategy of combing P-AML-5G with the MRD status after the initial induction therapy might help identify patients at a higher risk in the P-AML-5G low-risk group and prevent a worse prognosis. From the perspective of screening HSCT candidates, patients in the high-risk group identified by our model might benefit from HSCT, which is consistent with previous findings [30, 31]. Since the risk groups determined by P-AML-5G showed a significant 61% difference in survival probability in the TARGET discovery dataset. In the TARGET validation dataset, this difference was observed to be 44%. These findings highlight the potential relevance of P-AML-5G in guiding HSCT treatment decisions for patients in the intermediate-risk category.

Expression levels of five feature genes (*ZNF775*, *RNFT1*, *CRNDE*, *COL23A1*, and *TTC38*) in the model were found to be significantly correlated with the prognosis of the P-AML patients. We also observed intense increased expression of *CRNDE* and *COL23A1* in the bone marrow of patients, compared to that of healthy children. This novel discovery serves as additional evidence that reinforces the significance of these genes in pediatric AML. It expands the potential application of these genes in disease prognosis and underscores the importance of conducting further research on the functional mechanisms. *The Zinc Finger Protein 775 (ZNF775) has been predicted to enable the DNA-binding transcription factor activity and found to have strong predictive value for the OS in the hepatocellular carcinoma patients* [32], however, the functional role of this protein has not been explored in detail. Collagen Type XXIII Alpha 1 Chain (COL23A1) is reportedly associated with prostate cancer recurrence and distant metastases [33] and clinical stages in thyroid carcinoma [34]. Further investigation of urine samples from prostate cancer patients before and after prostatectomy suggested that collagen XXIII may have application as a biomarker in human fluids. Unfortunately, no reports on the function of the Ring Finger Protein, Transmembrane 1 (RNFT1), and Tetratricopeptide Repeat Domain 38 (TTC38) or their role in cancer have been found so far. Especially, *Colorectal neoplasia differentially expressed (CRNDE)* is a well-known long non-coding RNA which was considered to play crucial roles in the development of multiple cancers [35]. Moreover, it was highly expressed in AML [35, 36], displayed functional roles in AML proliferation [37] and indicated by previous analyses to have prognostic value in AML [36, 38]. Explorations focusing on the detailed mechanisms of *CRNDE* in P-AML pathology might help to identify a promising therapeutic strategy.

Taken together, we propose a robust P-AML-5G prognostic model specific for pediatric AML, which was created particularly utilizing data from pediatric AML outcomes. It has the potential to redefine traditional COG risk categorization, identify patients at high risk and offer the possibility of clinical application for the development of innovative treatment options by decreasing the panel and complexity of genetic markers without sacrificing the efficacy of the predictive capacity.

## Materials and methods

### Samples and datasets

Therapeutically Applicable Research to Generate Effective Treatments (TARGET) AML series consist of clinical data and RNA-sequencing from 256 peripheral blood or bone marrow samples of children, adolescents, and young adults with de novo AML enrolled on biology studies and clinical trials managed through the Children’s Oncology Group, hereafter referred to as the “TARGET 256”. OS is defined as the time from study entry until death. EFS is defined as the time from study entry until death, induction failure or relapse. Overall, the mean time of the follow-up was 4.15 ± 2.84 years for TARGET 256. A subset of samples from TARGET AML project was randomly chosen and provided to the Genomic Data Commons (GDC) database for harmonization (N = 145), facilitating integration and analysis of multiple datasets for GDC researchers. This dataset served as the training cohort for our study (https://portal.gdc.cancer.gov/projects/TARGET-AML, collected on 2021–10-21). The remaining 111 patients from TARGET were used as the validation set, simulating two distinct sets with clinical differences, akin to real-world scenarios of training and validation sets, gathered from the NCI’s data portal on 2021–10-21[https://target.nci.nih.gov/dataMatrix/TARGET_DataMatrix.html]. Individual-level whole-genome sequencing data of 150 samples in TARGET cohort were obtained from dbGaP (accession number phs000465). RNA-Sequencing and clinical data of 139 patients in Japan P-AML dataset deposited in the European Genome-Phenome Archive (EGAS00001003701) [39] was extracted for external validation purpose, hereafter referred to as the “JAPAN P-AML”. In addition, RNA-sequencing and clinical data from 943 pediatric patients and 70 normal bone marrow samples, obtained from the COG study AAML1031 [40], were collected for external validation (collected on 2023–11-03, 923 out of 943 patients with survival data available). These data, deposited in the GDC, were also utilized to examine the expression disparities of prognostic genes between patients and healthy children.

### Prognostic score construction and validation for AML

Combined with the survival information, the genes related to the prognosis of P-AML patients in TARGET 145 were screened out by univariate Cox proportional hazards regression analysis using normalized counts value from DESeq2 tool [41] (*p* < 0.05). LASSO regression analysis was conducted to identify the most stable gene set with 1000 time iterations [42]. Subsequently, Kaplan–Meier survival analysis and multivariable Cox regression analysis were performed and a prognostic risk score formula was established based on a linear combination of expression levels weighted with the regression coefficients derived from the multivariate logistic regression analysis. Risk score = expression of gene 1 × *β*1 + expression of gene 2 × *β*2 + ⋯ + expression of gene *n* × *βn*. *β* values are the regression coefficients derived from the multivariate logistic regression analysis of the dataset. The optimal cutoff of risk score was determined via the maxstat package in R [43, 44], dividing patients into high-risk group and low-risk groups. TARGET 111 and JAPAN P-AML were used for prognostic value validation. All patients in the database were scored using the formula, and the optimal cutoff risk score was used to divide the patients into high and low groups. Kaplan–Meier survival analysis was applied to assess the prognostic value of the derived risk groups.

The traditional COG risk system used in the clinical trials on which the TARGET patients were enrolled (COG) [9, 45], expanded COG AAML1831 risk stratification system (expanded_COG_AAML1831) [16], 2022 ELN risk stratification system (2022ELN) [18] and two leukemic stem cell (LSC) score based models established for adult (LSC17) [46] or pediatric (LSC6) [14] AML, were selected for model comparison (references and detailed models can be found in Table S2; definition of risk-groups for COG, expanded_COG_AAML1831 and 2022ELN Classification systems can be found in Table S3).

### Statistical analysis

The chi-square test was applied for comparing the statistical difference in categorical variables, and two-tailed Student's *t* test or Wilcoxon test was used for quantitative variables. Kaplan–Meier curves were plotted to estimate overall survival, and the log-rank test was performed to evaluate statistical significance of differences in survival. Variables identified as significant factors in the univariate Cox analysis were selected into the multivariate Cox proportional hazards regression analysis to identify the independent prognostic factors using the Forward Stepwise (conditional LR) method. ROC curves (receiver operating characteristic curves), the area under the curve (AUC) and the Harrell's concordance index (C-index) [47] were utilized with survival package in R to determine predictive values. Differentially expressed genes (DEGs) between pediatric patients and normal bone marrow samples in COG AAML1031 study were analyzed with R package DESeq2 [41]. All statistical analyses were carried out using R (3.5.2) software, and *p* < 0.05 (bilateral) was defined as a statistical difference.

### Supplementary Information

**Additional file 1:** **Tables S1.** Clinical characteristics of the P-AML patients. **Tables S2.** Gene and coefficient list of P-AML-5G and two LSC models established for adult or pediatric AML. **Tables S3.** Definition of risk classification systems for P-AML (COG and expanded-COG-AAML1831) and adult AML (2022ELN). **Fig. S1.** Kaplan-Meier curves of overall survival (OS, days) based on three treatment subgroups in TARGET 256. **Fig. S2**. Kaplan-Meier survival analysis for the evaluation of clinical potentials of 14 target genes. **Fig. S3. **(a) Kaplan-Meier curves of EFS based on risk-groups defined by P-AML-5G prognosis model (*p*<0.001); (b) ROC analysis of P-AML-5G score for prediction of EFS risk at 1, 3, and 5 years in TARGET 145 cohort. **Fig. S4.** Expression levels of 5 genes for constructing the P-AML-5G model were used to compare the groups. ****p<0.0001 from Wilcoxon rank sum test. **Fig. S5.** Risk group stratification of P-AML-5G in the treatment subgroups of AAML03P1 (a), AAML0531 (b) and CCG2961 (c) in TARGET 145. **Fig. S6. **Kaplan-Meier curves of EFS based on risk-groups defined by P-AML-5G prognosis model in TARGET validation (*p*<0.001). **Fig. S7.** Gene expression for each of the genes in the P-AML-5G model in patient and healthy control groups of AAML1031 study. Data are expressed as the normalized counts from Deseq2 analysis. ****p<0.0001 and log2(foldchange)>3 from Deseq2 analysis. **Fig. S8.** Sankey diagram of the P-AML-5G and COG risk groups in (a) TARGET 145 and (b) TARGET validation. Risk groups are illustrated by colored boxes. Middle areas indicate case redistribution flow. **Fig. S9.** Kaplan-Meier curves for overall survival (OS) of risk groups in TARGET 256 cohort stratified by MRD1 and HSCT status. (a) Kaplan–Meier curves for OS of patients with and without Minimal Residual Disease At End the First Course (MRD1), (b) Stem Cell Transplant During First Complete Remission (HSCT). **Fig. S10.** Kaplan-Meier curves of OS (a) and EFS (b) based on risk-groups defined by P-AML-5G prognosis model in peripheral blood subgroup of TARGET 256 (*N*=42).

## Data Availability

TARGET 145 RNA-sequencing is available for download through the Broad Institute GDAC Firehose repository [https://gdac.broadinstitute.org/]. Clinical and RNA-sequencing data for TARGET generated for this study have been collected from TARGET Data Matrix at the TARGET Data Coordinating Center [https://target.nci.nih.gov/dataMatrix/TARGET_DataMatrix.html]. Clinical and RNA-sequencing data for AAML1031 study was collected from GDC Data Portal (https://portal.gdc.cancer.gov/). Whole genome-sequencing data were applied from TARGET controlled-access datasets (dbGaP Study Accession: phs000218.v4.p1) with the approvement of the database of Genotypes and Phenotypes (dbGaP) (https://www.ncbi.nlm.nih.gov/gap/). Data used for model validation in JAPAN P-AML dataset can be applied from The European Genome-phenome Archive (EGA) (https://ega-archive.org/datasets/EGAD00001005078).
