# Supplementary material for: Development and validation of a promising 5-gene prognostic model for pediatric acute myeloid leukemia
Source: Mol Biomed. 2024 Jan 2;5:1. doi: 10.1186/s43556-023-00162-y (PMC10758381; doi:10.1186/s43556-023-00162-y)
Supplement: Supplementary file 1 — Additional file 1: Tables S1. Clinical characteristics of the P-AML patients. Tables S2. Gene and coefficient list of P-AML-5G and two LSC models established for adult or pediatric AML. Tables S3. Definition of risk classification systems for P-AML (COG and expanded-COG-AAML1831) and adult AML (2022ELN). Fig. S1. Kaplan-Meier curves of overall survival (OS, days) based on three treatment subgroups in TARGET 256. Fig. S2. Kaplan-Meier survival analysis for the evaluation of clinical potentials of 14 target genes. Fig. S3. (a) Kaplan-Meier curves of EFS based on risk-groups defined by P-AML-5G prognosis model (p<0.001); (b) ROC analysis of P-AML-5G score for prediction of EFS risk at 1, 3, and 5 years in TARGET 145 cohort. Fig. S4. Expression levels of 5 genes for constructing the P-AML-5G model were used to compare the groups. ****p<0.0001 from Wilcoxon rank sum test. Fig. S5. Risk group stratification of P-AML-5G in the treatment subgroups of AAML03P1 (a), AAML0531 (b) and CCG2961 (c) in TARGET 145. Fig. S6. Kaplan-Meier curves of EFS based on risk-groups defined by P-AML-5G prognosis model in TARGET validation (p<0.001). Fig. S7. Gene expression for each of the genes in the P-AML-5G model in patient and healthy control groups of AAML1031 study. Data are expressed as the normalized counts from Deseq2 analysis. ****p<0.0001 and log2(foldchange)>3 from Deseq2 analysis. Fig. S8. Sankey diagram of the P-AML-5G and COG risk groups in (a) TARGET 145 and (b) TARGET validation. Risk groups are illustrated by colored boxes. Middle areas indicate case redistribution flow. Fig. S9. Kaplan-Meier curves for overall survival (OS) of risk groups in TARGET 256 cohort stratified by MRD1 and HSCT status. (a) Kaplan–Meier curves for OS of patients with and without Minimal Residual Disease At End the First Course (MRD1), (b) Stem Cell Transplant During First Complete Remission (HSCT). Fig. S10. Kaplan-Meier curves of OS (a) and EFS (b) based on risk-groups defined by P-AML-5G prognosis [file 43556_2023_162_MOESM1_ESM.pdf]

Development and validation of a promising 5-gene prognostic model for pediatric acute myeloid leukemia

Running Title: Five-gene prognostic model for P-AML

Yu Tao[1#], Li Wei[2,3#], Norio Shiba[4], Daisuke Tomizawa[5],

Yasuhide Hayashi[6], Seishi Ogawa[7,8,9], Li Chen[10], Hua You, MD[1*]

[1]Laboratory for Excellence in Systems Biomedicine of Pediatric Oncology, Department of Pediatric Hematology and Oncology, Chongqing Key Laboratory of Pediatrics, Ministry of Education Key Laboratory of Child Development and Disorders, China International Science and Technology Cooperation base of Child development and Critical Disorders, National Clinical Research Center for Child Health and Disorders, Children's Hospital of Chongqing Medical University, Chongqing, China

[2]NHC Key Laboratory of Birth Defects and Reproductive Health, Chongqing Population and Family Planning Science and Technology Research Institute, Chongqing, China

[3]Chongqing Hospital of Traditional Chinese Medicine, Chongqing, China

[4]Department of pediatrics, Yokohama City University Graduate School of Medicine, Yokohama, Japan

[5]Division of Leukemia and Lymphoma, Children's Cancer Center, National Center for Child Health and Development, Tokyo, Japan

[6]Department of Hematology/Oncology, Gunma Children's Medical Center, Gunma and Institute of Physiology and Medicine, Jobu University, Gunma, Japan

[7]Department of Pathology and Tumor Biology, Kyoto University, Kyoto, Japan

[8]Institute for the Advanced Study of Human Biology (WPI–ASHBi), Kyoto University, Kyoto, Japan

[9]Department of Medicine, Center for Hematology and Regenerative Medicine, Karolinska Institute, Stockholm 17177, Sweden

[10]Department of Cellular and Genetic Medicine, School of Basic Medical Sciences, Fudan University, Shanghai 200032, China

#: These authors contributed equally.

*: Corresponding author: Hua You, M.D., Ph.D.

Laboratory for Excellence in Systems Biomedicine of Pediatric Oncology, Department of Pediatric Hematology and Oncology, Chongqing Key Laboratory of Pediatrics, Ministry of Education Key Laboratory of Child Development and Disorders, China International Science and Technology Cooperation base of Child development and Critical Disorders, National Clinical Research Center for Child Health and Disorders, Children's Hospital of Chongqing Medical University, 136 Zhongshaner Rd, Yuzhong District, Chongqing, 401122, China.

Email: youhua307@163.com

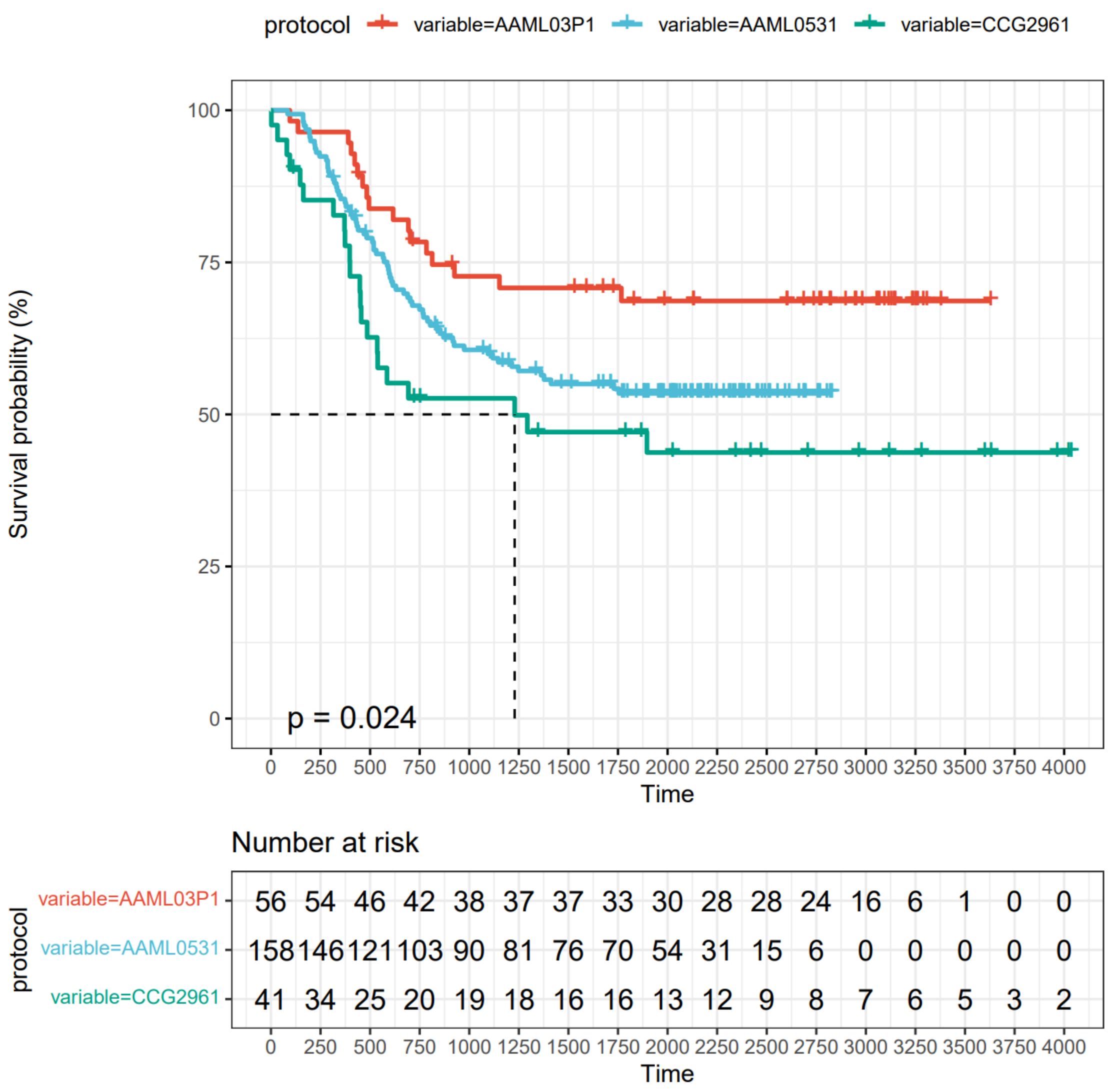

Fig.S1. Kaplan-Meier curves of overall survival (OS, days) based on three treatment subgroups in TARGET 256.

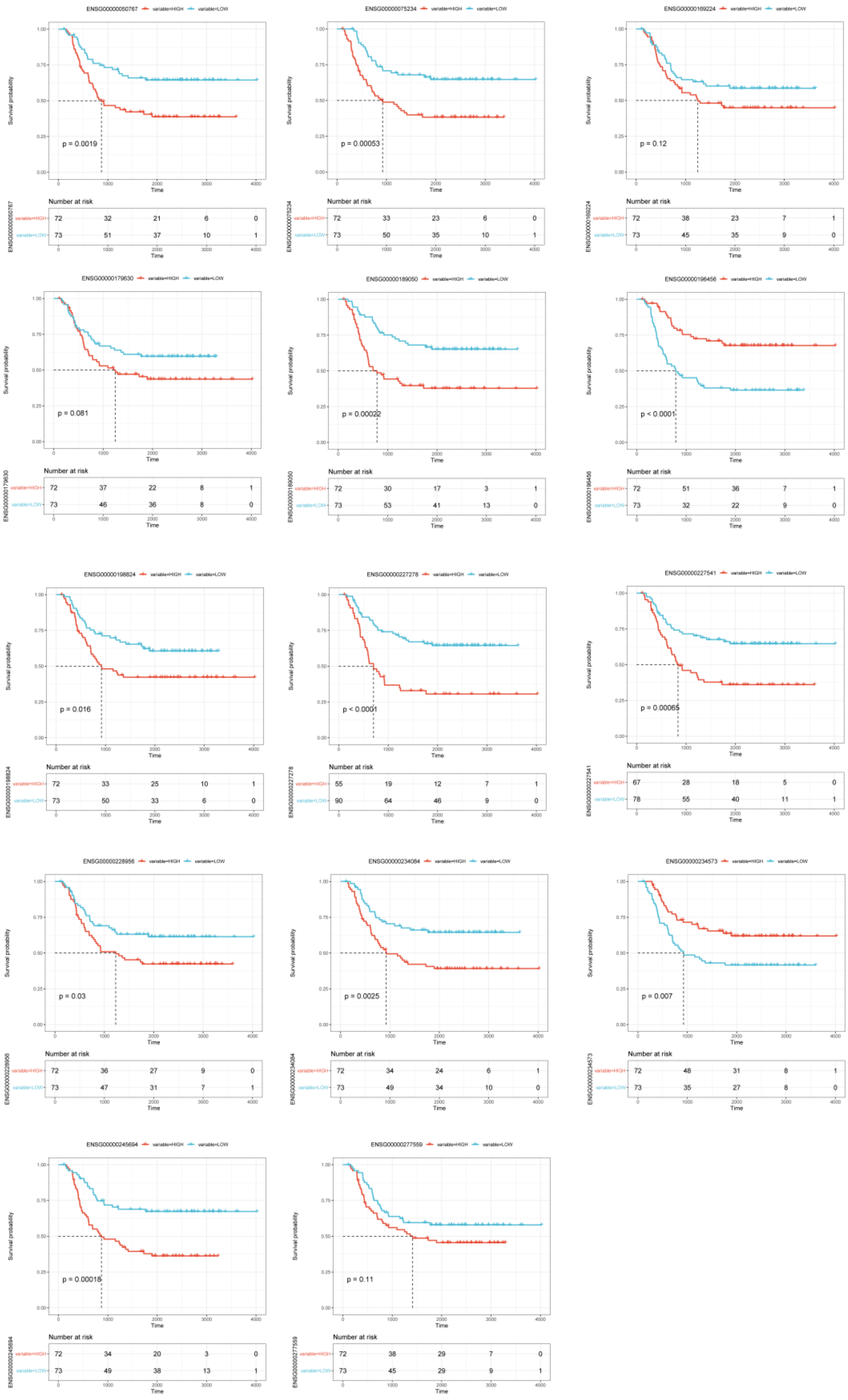
ENSG00000050767
p = 0.0019
ENSG00000075234
p = 0.00053
ENSG00000169224
p = 0.12
ENSG00000179630
p = 0.081
ENSG00000189050
p = 0.00022
ENSG00000196456
p < 0.0001
ENSG00000198824
p = 0.016
ENSG00000227278
p < 0.0001
ENSG00000227541
p = 0.00065
ENSG00000228956
p = 0.03
ENSG00000234084
p = 0.0025
ENSG00000234573
p = 0.007
ENSG00000245694
p = 0.00018
ENSG00000277559
p = 0.11

Fig.S2. Kaplan-Meier survival analysis for the evaluation of clinical potentials of 14 target genes.

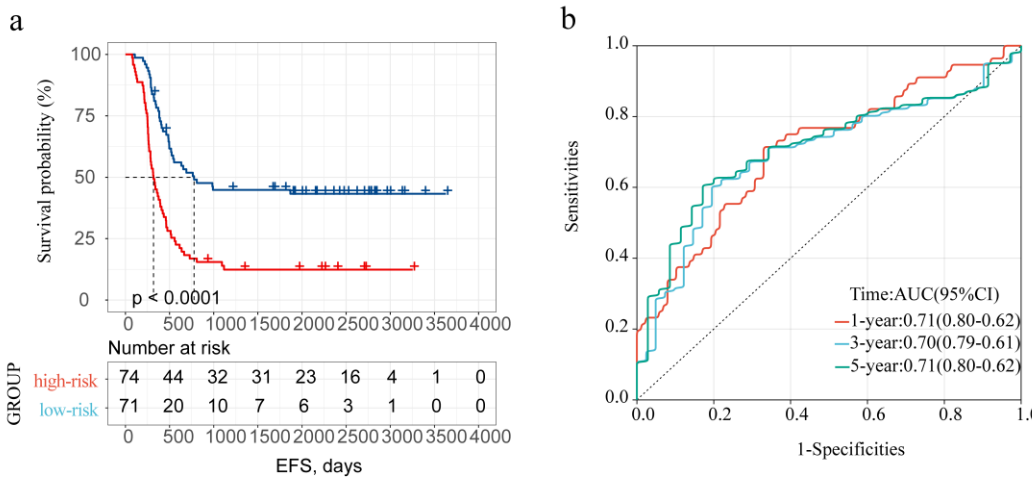

Fig.S3. (a) Kaplan-Meier curves of EFS based on risk-groups defined by P-AML-5G prognosis model (*p*<0.001); (b) ROC analysis of P-AML-5G score for prediction of EFS risk at 1, 3, and 5 years in TARGET 145 cohort.

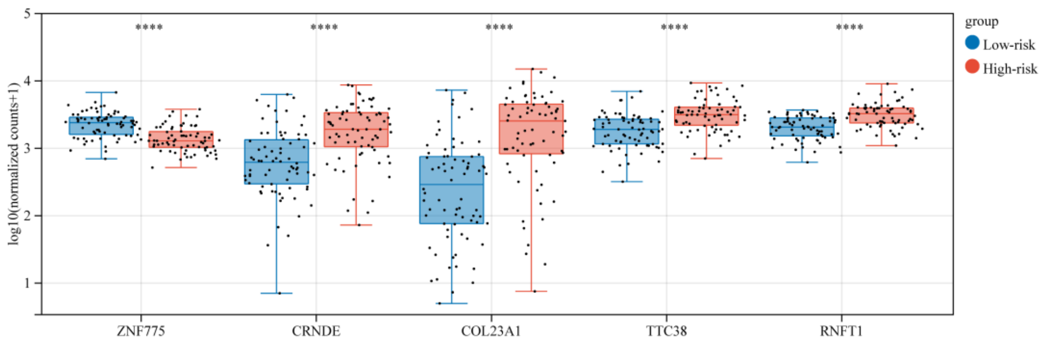

Fig.S4. Expression levels of 5 genes for constructing the P-AML-5G model were used to compare the groups. ****p<0.0001 from Wilcoxon rank sum test.

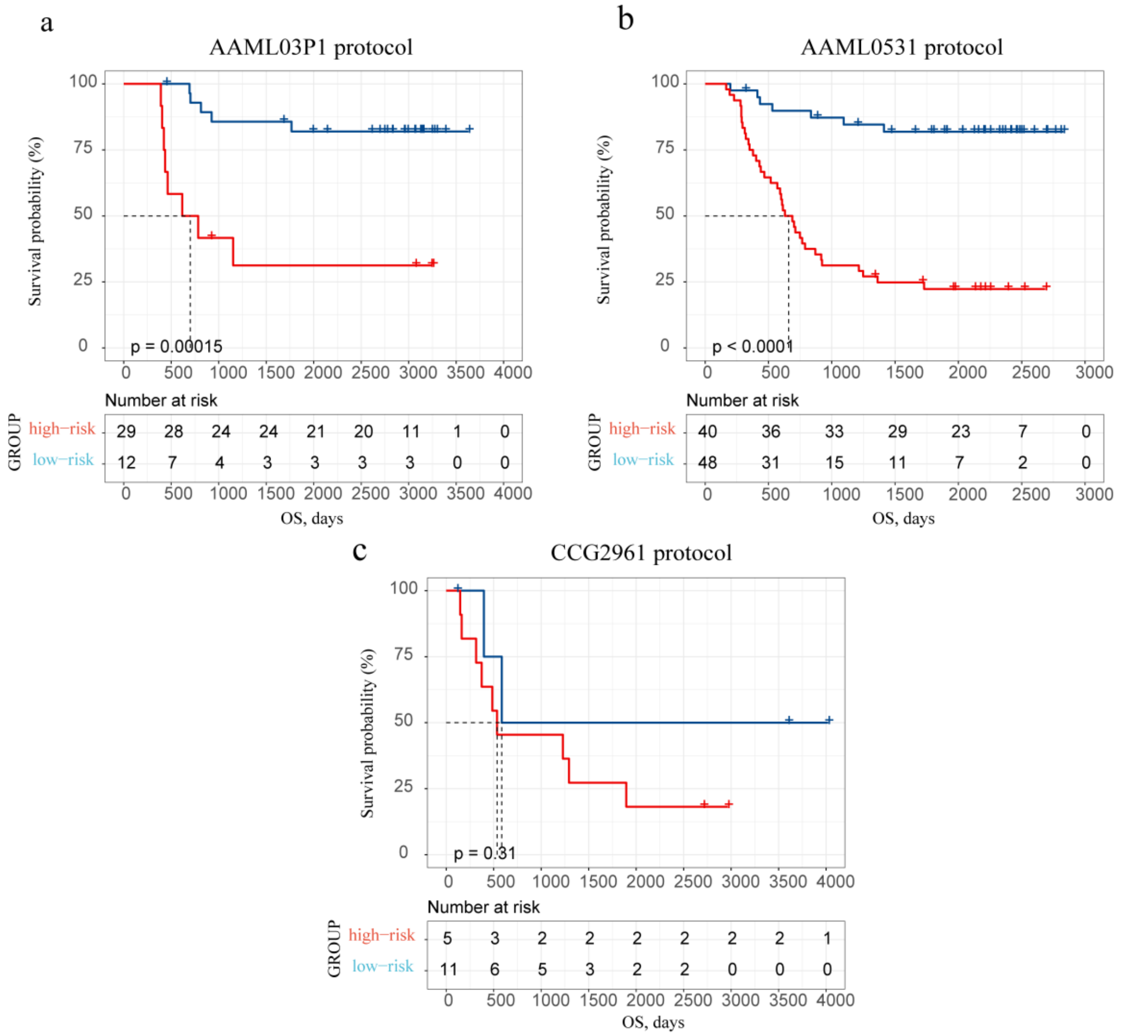

Fig.S5. Risk group stratification of P-AML-5G in the treatment subgroups of AAML03P1 (a), AAML0531 (b) and CCG2961 (c) in TARGET 145.

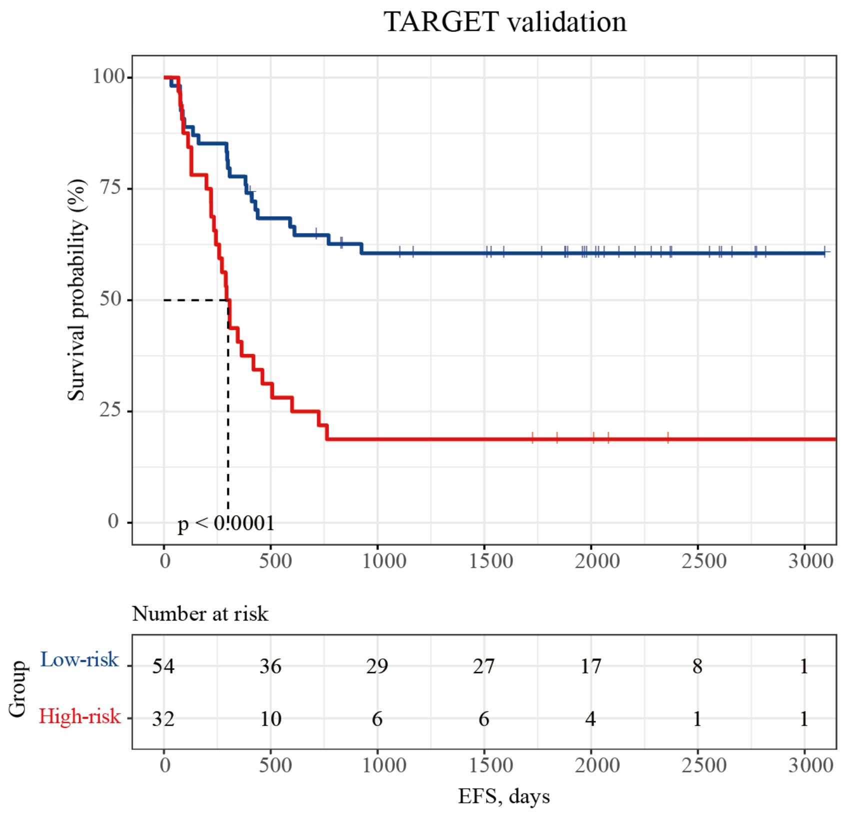

Fig.S6. Kaplan-Meier curves of EFS based on risk-groups defined by P-AML-5G prognosis model in TARGET validation (p<0.001).

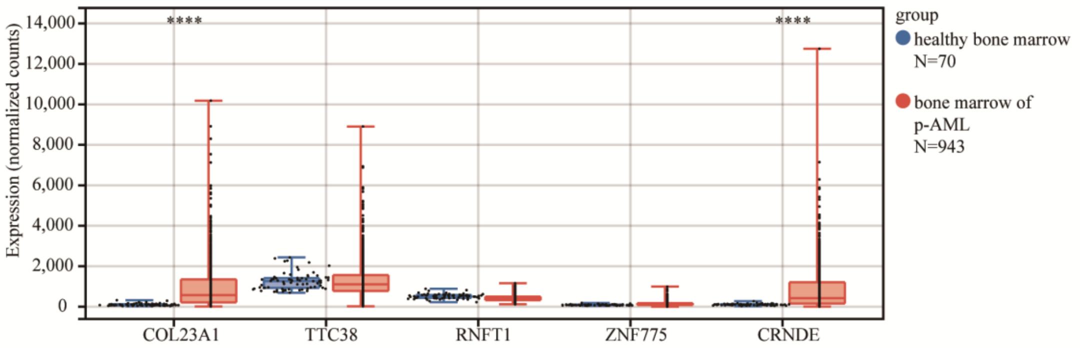

Fig.S7. Gene expression for each of the genes in the P-AML-5G model in patient and healthy control groups of AAML1031 study. Data are expressed as the normalized counts from Deseq2 analysis. ****p<0.0001 and log2(foldchange)>3 from Deseq2 analysis.

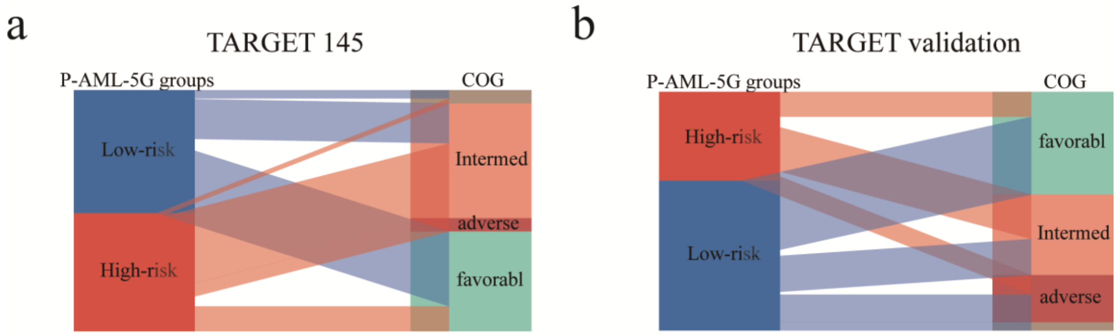

Fig.S8. Sankey diagram of the P-AML-5G and COG risk groups in (a) TARGET 145 and (b) TARGET validation. Risk groups are illustrated by colored boxes. Middle areas indicate case redistribution flow.

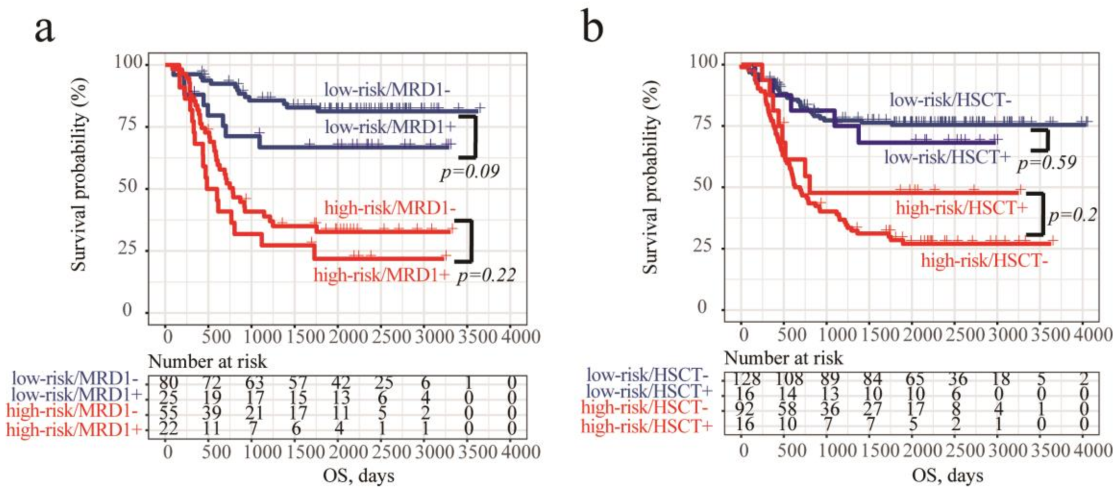

Fig.S9. Kaplan-Meier curves for overall survival (OS) of risk groups in TARGET 256 cohort stratified by MRD1 and HSCT status. (a) Kaplan–Meier curves for OS of patients with and without Minimal Residual Disease At End the First Course (MRD1), (b) Stem Cell Transplant During First Complete Remission (HSCT).

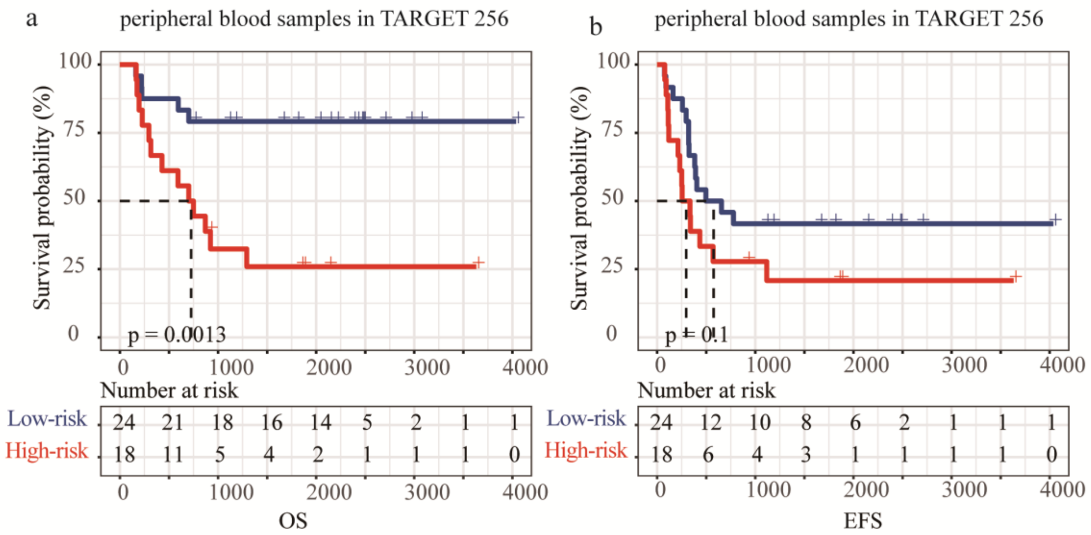

Fig.S10. Kaplan-Meier curves of OS (a) and EFS (b) based on risk-groups defined by P-AML-5G prognosis model in peripheral blood subgroup of TARGET 256 (N=42).

Tables S1. Clinical characteristics of the P-AML patients

**Characteristic**	**TARGET 145** N = 145[1]	**TARGET 111** N = 111[1]	**TARGET 256** N = 256[1]	**p-value**[2]	**COG AAML1031 N=923***	**Japan P-AML** N=139[1]
Gender				0.534		
Female	71 (48.97%)	50 (45.05%)	121 (47.27%)		445(48.21%)	69(49.64%)
Male	74 (51.03%)	61 (54.95%)	135 (52.73%)		478(51.79%)	70(50.36%)
Race				0.165		-
American Indian or Alaska Native	1 (0.69%)	0 (0.00%)	1 (0.39%)		7(0.76%)	
Asian	3 (2.07%)	4 (3.60%)	7 (2.73%)		45(4.88%)	
Black or African American	15 (10.34%)	13 (11.71%)	28 (10.94%)		107(11.59%)	
Native Hawaiian or other Pacific Islander	3 (2.07%)	0 (0.00%)	3 (1.17%)		5(0.54%)	
White	112 (77.24%)	77 (69.37%)	189 (73.83%)		99(10.73%)	
Unknown	11 (7.59%)	17 (15.32%)	28 (10.94%)		7(0.76%)	
Ethnicity				0.439		-
Hispanic or Latino	26 (17.93%)	23 (20.72%)	49 (19.14%)		162(17.55%)	
Not Hispanic or Latino	115 (79.31%)	82 (73.87%)	197 (76.95%)		730(79.09%)	
Unknown	4 (2.76%)	6 (5.41%)	10 (3.91%)		31(3.36%)	
Age at Diagnosis, years	9.22±6.06	10.35±5.43	9.71±5.81	0.118	9.96±6.45	8.80±0.21
Age Group, years				0.132		-
<=3	37 (25.52%)	17 (15.32%)	54 (21.09%)		62(6.72%)	
3~14	77 (53.10%)	69 (62.16%)	146 (57.03%)		612(66.31%)	
>=15	31 (21.38%)	25 (22.52%)	56 (21.88%)		249(26.98%)	
First Event				<0.001		-
Censored	42 (28.97%)	46 (41.44%)	88 (34.38%)		435(47.13%)	
Death	1 (0.69%)	3 (2.70%)	4 (1.56%)		51(5.53%)	
Death without remission	0 (0.00%)	5 (4.50%)	5 (1.95%)		15(1.63%)	
Induction failure	3 (2.07%)	7 (6.31%)	10 (3.91%)		65(7.04%)	
Relapse	99 (68.28%)	50 (45.05%)	149 (58.20%)		357(38.68%)	
EFS, years	2.62±2.66	2.90±2.80	2.74±2.72	0.411	2.42±2.00	2.03±5.20
OS, years	4.32±2.91	3.96±2.76	4.17±2.85	0.318	3.21±1.93	2.50±5.02
Vital status				0.192		
Alive	77(53.10%)	68(61.26%)	145(56.64%)		620(67.17%)	87(62.59%)
Dead	68(46.90%)	43(38.74%)	111(43.36%)		303(32.83%)	52(37.41%)
Protocol				0.003	-	-
AAML03P1	41 (28.28%)	15 (13.51%)	56 (21.88%)			

Characteristic	TARGET 145<br>N = 145[1]	TARGET 111<br>N = 111[1]	TARGET 256<br>N = 256[1]	p-value[2]	COG AAML1031<br>N=923*	Japan P-AML<br>N=139[1]
AAML0531	88 (60.69%)	71 (63.96%)	159 (62.11%)			
CCG2961	16 (11.03%)	25 (22.52%)	41 (16.02%)			
WBC at diagnosis,10^3 mcl				0.633	-	-
Median (IQR)	45 (17, 95)	43 (18, 102)	45 (17, 95)			
Range	1, 519	1, 432	1, 519			
BM at diagnosis, %				0.467	-	-
Median (IQR)	75 (60, 89)	72 (56, 88)	73 (58, 89)			
Range	14, 100	25, 98	14, 100			
Unknown	3	4	7			
PM at diagnosis, %				0.318	-	-
Median (IQR)	61 (37, 78)	61 (44, 83)	61 (40, 80)			
Range	0, 97	0, 97	0, 97			
CNS disease				0.533	-	-
No	135(93.10%)	101(90.99%)	236(92.20%)			
Yes	10(6.90%)	10(9.01%)	20(7.80%)			
Chloroma				0.016	-	-
No	134 (92.41%)	92 (82.88%)	226 (88.28%)			
Unknown	1 (0.69%)	0 (0.00%)	1 (0.39%)			
Yes	10 (6.90%)	19 (17.12%)	29 (11.33%)			
FAB Category				-	-	-
M0 Undifferentiated	3 (2.07%)	0 (0.00%)	3 (1.17%)			
M1	17 (11.72%)	16 (14.41%)	33 (12.89%)			
M2	35 (24.14%)	31 (27.93%)	66 (25.78%)			
M4	36 (24.83%)	26 (23.42%)	62 (24.22%)			
M5	30 (20.69%)	19 (17.12%)	49 (19.14%)			
M6	2 (1.38%)	1 (0.90%)	3 (1.17%)			
M7	7 (4.83%)	0 (0.00%)	7 (2.73%)			7(5.04%)
Not classified	8 (5.52%)	6 (5.41%)	14 (5.47%)			
Unknown	7 (4.83%)	12 (10.81%)	19 (7.42%)			
c6_9*				0.771	-	-
No	134 (92.41%)	102 (91.89%)	236 (92.19%)			
Unknown	10 (6.90%)	7 (6.31%)	17 (6.64%)			
Yes	1 (0.69%)	2 (1.80%)	3 (1.17%)			
c8_21				0.423	-	-
No	114 (78.62%)	81 (72.97%)	195 (76.17%)			

Characteristic		TARGET 145<br>N = 145[1]	TARGET 111<br>N = 111[1]	TARGET 256<br>N = 256[1]	p-value[2]	COG AAML1031<br>N=923*	Japan P-AML<br>N=139[1]
	Unknown	10 (6.90%)	7 (6.31%)	17 (6.64%)			
	Yes	21 (14.48%)	23 (20.72%)	44 (17.19%)			
t(3;5)(q25;q34)*					>0.999	-	-
	No	133 (91.72%)	103 (92.79%)	236 (92.19%)			
	Unknown	10 (6.90%)	7 (6.31%)	17 (6.64%)			
	Yes	2 (1.38%)	1 (0.90%)	3 (1.17%)			
t(6;11)(q27;q23) *					>0.999	-	-
	No	133 (91.72%)	102 (91.89%)	235 (91.80%)			
	Unknown	10 (6.90%)	7 (6.31%)	17 (6.64%)			
	Yes	2 (1.38%)	2 (1.80%)	4 (1.56%)			
t(9;11)(p22;q23)					0.375	-	-
	No	121 (83.45%)	98 (88.29%)	219 (85.55%)			
	Unknown	11 (7.59%)	8 (7.21%)	19 (7.42%)			
	Yes	13 (8.97%)	5 (4.50%)	18 (7.03%)			
t(10;11)(p11.2;q23) *					0.941	-	-
	No	131 (90.34%)	102 (91.89%)	233 (91.02%)			
	Unknown	10 (6.90%)	7 (6.31%)	17 (6.64%)			
	Yes	4 (2.76%)	2 (1.80%)	6 (2.34%)			
t(11:19)(q23:p13.1) *					0.164	-	-
	No	130 (89.66%)	104 (93.69%)	234 (91.41%)			
	Unknown	10 (6.90%)	7 (6.31%)	17 (6.64%)			
	Yes	5 (3.45%)	0 (0.00%)	5 (1.95%)			
inv(16)					0.335	-	-
	No	107 (73.79%)	90 (81.08%)	197 (76.95%)			
	Unknown	10 (6.90%)	7 (6.31%)	17 (6.64%)			
	Yes	28 (19.31%)	14 (12.61%)	42 (16.41%)			
del5q*					>0.999	-	-
	No	134 (92.41%)	104 (93.69%)	238 (92.97%)			
	Unknown	10 (6.90%)	7 (6.31%)	17 (6.64%)			
	Yes	1 (0.69%)	0 (0.00%)	1 (0.39%)			
del7q*					0.767	-	-
	No	131 (90.34%)	99 (89.19%)	230 (89.84%)			
	Unknown	10 (6.90%)	7 (6.31%)	17 (6.64%)			

Characteristic		TARGET 145 N = 145[1]	TARGET 111 N = 111[1]	TARGET 256 N = 256[1]	p-value[2]	COG AAML1031 N=923*	Japan P-AML N=139[1]
	Yes	4 (2.76%)	5 (4.50%)	9 (3.52%)			
del9q					0.904	-	-
	No	130 (89.66%)	99 (89.19%)	229 (89.45%)			
	Unknown	10 (6.90%)	7 (6.31%)	17 (6.64%)			
	Yes	5 (3.45%)	5 (4.50%)	10 (3.91%)			
monosomy5*					0.851	-	-
	No	135 (93.10%)	104 (93.69%)	239 (93.36%)			
	Unknown	10 (6.90%)	7 (6.31%)	17 (6.64%)			
monosomy7*					0.692	-	
	No	135 (93.10%)	103 (92.79%)	238 (92.97%)			136(97.84%)
	Unknown	10 (6.90%)	7 (6.31%)	17 (6.64%)			
	Yes	0 (0.00%)	1 (0.90%)	1 (0.39%)			3(2.16%)
trisomy8					0.832	-	
	No	126 (86.90%)	95 (85.59%)	221 (86.33%)			121(86.33%)
	Unknown	10 (6.90%)	7 (6.31%)	17 (6.64%)			
	Yes	9 (6.21%)	9 (8.11%)	18 (7.03%)			18(13.67%)
trisomy21*					0.662	-	-
	No	131 (90.34%)	103 (92.79%)	234 (91.41%)			
	Unknown	10 (6.90%)	7 (6.31%)	17 (6.64%)			
	Yes	4 (2.76%)	1 (0.90%)	5 (1.95%)			
MLL					0.483	-	-
	No	112 (77.24%)	92 (82.88%)	204 (79.69%)			
	Unknown	10 (6.90%)	7 (6.31%)	17 (6.64%)			
	Yes	23 (15.86%)	12 (10.81%)	35 (13.67%)			
MinusY					>0.999	-	-
	No	129 (88.97%)	99 (89.19%)	228 (89.06%)			
	Unknown	10 (6.90%)	7 (6.31%)	17 (6.64%)			
	Yes	6 (4.14%)	5 (4.50%)	11 (4.30%)			
MinusX					>0.999	-	-
	No	129 (88.97%)	100 (90.09%)	229 (89.45%)			
	Unknown	10 (6.90%)	7 (6.31%)	17 (6.64%)			
	Yes	6 (4.14%)	4 (3.60%)	10 (3.91%)			
Cytogenetic Complexity					0.757	-	-

Characteristic	TARGET 145 N = 145[1]	TARGET 111 N = 111[1]	TARGET 256 N = 256[1]	p-value[2]	COG AAML1031 N=923*	Japan P-AML N=139[1]
0~2	115 (79.31%)	91 (81.98%)	206 (80.47%)			
3andmore	23 (15.86%)	14 (12.61%)	37 (14.45%)			
Unknown	7 (4.83%)	6 (5.41%)	13 (5.08%)			
Primary Cytogenetic Code				0.157	-	-
Normal	27 (18.62%)	32 (28.83%)	59 (23.05%)			
notnormal	108 (74.48%)	72 (64.86%)	180 (70.31%)			
Unknown	10 (6.90%)	7 (6.31%)	17 (6.64%)			
FLT3_ITD				<0.001	-	
No	134(92.41%)	82(73.87%)	216(84.38%)			106(76.26%)
Yes	11(7.59%)	29(26.13%)	40(15.63%)			33(23.74%)
FLT3_PM				0.290	-	-
No	134 (92.41%)	102 (91.89%)	236 (92.19%)			
Unknown	0 (0.00%)	2 (1.80%)	2 (0.78%)			
Yes	11 (7.59%)	7 (6.31%)	18 (7.03%)			
NPM				0.030	-	
No	135 (93.10%)	93 (83.78%)	228 (89.06%)			123(88.49%)
Unknown	5 (3.45%)	5 (4.50%)	10 (3.91%)			
Yes	5 (3.45%)	13 (11.71%)	18 (7.03%)			21(15.115)
CEBPA				0.234	-	-
No	137 (94.48%)	99 (89.19%)	236 (92.19%)			
Unknown	1 (0.69%)	3 (2.70%)	4 (1.56%)			
Yes	7 (4.83%)	9 (8.11%)	16 (6.25%)			
WT1				0.673	-	-
No	133 (91.72%)	98 (88.29%)	231 (90.23%)			
Unknown	4 (2.76%)	4 (3.60%)	8 (3.13%)			
Yes	8 (5.52%)	9 (8.11%)	17 (6.64%)			
KIT_EXON8*				0.679	-	-
No	33 (22.76%)	28 (25.23%)	61 (23.83%)			
Unknown	102 (70.34%)	78 (70.27%)	180 (70.31%)			
Yes	10 (6.90%)	5 (4.50%)	15 (5.86%)			
KIT_EXON17*				0.185	-	-
No	32 (22.07%)	30 (27.03%)	62 (24.22%)			
Unknown	102 (70.34%)	78 (70.27%)	180 (70.31%)			

Characteristic	TARGET 145<br>N = 145[1]	TARGET 111<br>N = 111[1]	TARGET 256<br>N = 256[1]	p-value[2]	COG AAML1031 N=923*	Japan P-AML<br>N=139[1]
Yes	11 (7.59%)	3 (2.70%)	14 (5.47%)			
MRD1				0.140	-	-
No	83 (57.24%)	52 (46.85%)	135 (52.73%)			
Unknown	35 (24.14%)	39 (35.14%)	74 (28.91%)			
Yes	27 (18.62%)	20 (18.02%)	47 (18.36%)			
CR1				0.421	-	-
CR	127 (87.59%)	92 (82.88%)	219 (85.55%)			
death	0 (0.00%)	2 (1.80%)	2 (0.78%)			
not in CR	17 (11.72%)	16 (14.41%)	33 (12.89%)			
unevaluable	1 (0.69%)	1 (0.90%)	2 (0.78%)			
COG				0.003		-
favorabl	60 (41.38%)	52 (46.85%)	112 (43.75%)		347(37.59%)	
Intermed	69 (47.59%)	34 (30.63%)	103 (40.23%)		465(50.38%)	
adverse	8 (5.52%)	20 (18.02%)	28 (10.94%)		102(11.05%)	
Unknown	8 (5.52%)	5 (4.50%)	13 (5.08%)		9(0.98%)	
HSCT				0.053	-	-
No	131 (90.34%)	89 (80.18%)	220 (85.94%)			
Unknown	1 (0.69%)	3 (2.70%)	4 (1.56%)			
Yes	13 (8.97%)	19 (17.12%)	32 (12.50%)			
Gemtuzumab ozogamicin treatment				0.004	-	-
Gentuzumab ozogamicin treatment	90 (62.07%)	47 (42.34%)	137 (53.52%)			
NO Gentuzumab ozogamicin treatment	39 (26.90%)	39 (35.14%)	78 (30.47%)			
Unknown	16 (11.03%)	25 (22.52%)	41 (16.02%)			
2022ELN*				<0.001	-	-
adverse	31 (21.38%)	2 (1.80%)	33 (12.89%)			
favorabl	56 (38.62%)	6 (5.41%)	62 (24.22%)			
intermed	57 (39.31%)	7 (6.31%)	64 (25.00%)			
Unknown	1 (0.69%)	96 (86.49%)	97 (37.89%)			
expanded_COG_AAML1831*				<0.001	-	-
favorabl	55 (37.93%)	6 (5.41%)	61 (23.83%)			
intermed	44 (30.34%)	2 (1.80%)	46 (17.97%)			
adverse	45 (31.03%)	7 (6.31%)	52 (20.31%)			
Unknown	1 (0.69%)	96 (86.49%)	97 (37.89%)			

Characteristic		TARGET 145<br>N = 145[1]	TARGET 111<br>N = 111[1]	TARGET 256<br>N = 256[1]	p-value[2]	COG AAML1031<br>N=923*	Japan P-AML<br>N=139[1]
NUP98 fusion					0.127	-	-
	No	100 (68.97%)	65 (58.56%)	165 (64.45%)			
	Yes	12 (8.28%)	8 (7.21%)	20 (7.81%)			
	Unknown	33 (22.76%)	38 (34.23%)	71 (27.73%)			
KMT2A-r					0.027	-	-
	No	79 (54.48%)	60 (54.05%)	139 (54.30%)			
	Yes	33 (22.76%)	13 (11.71%)	46 (17.97%)			
	Unknown	33 (22.76%)	38 (34.23%)	71 (27.73%)			
RUNX1-RUNX1T1					0.040	-	-
	No	88 (60.69%)	50 (45.05%)	138 (53.91%)			
	Yes	24 (16.55%)	23 (20.72%)	47 (18.36%)			
	Unknown	33 (22.76%)	38 (34.23%)	71 (27.73%)			
CBFB-MYH11					0.100	-	-
	not	84 (57.93%)	58 (52.25%)	142 (55.47%)			
	CBFB-MYH11	28 (19.31%)	15 (13.51%)	43 (16.80%)			
	Unknown	33 (22.76%)	38 (34.23%)	71 (27.73%)			

* 923 out of 943 patients have clinical data available.

[1]n (%); Mean (SD)

[2] (TARGET 145 vs. TARGET 111) Pearson's Chi-squared test; Fisher's exact test; Welch Two Sample t-test; Wilcoxon rank sum test

[*] Not included in univariate Cox regression analysis due to the rareness (carriers less than 3% in TARGET 145 or TARGET 111) or high rate of missing data (>50%)

Notes: EFS: event free survival time, time to first event (or censoring); OS: overall survival time, The number of years after diagnosis to the last follow-up or death of the patient; WBC: The absolute peripheral white blood cell count; BM: Bone Marrow Blast Cell Outcome Percentage Value; PM: Peripheral Blast Cell Outcome Percentage Value; Chloroma: Chloroma Disease At Diagnosis Present; CNS: Central Nervous System Disease At Diagnosis Present ; FAB: Leukemia French American British Morphology Code; c6_9：Chromosomal translocation between chromosome 6 and chromosome 9 present; c8_21：Chromosomal translocation between chromosome 8 and chromosome 21present; t(3;5)(q25;q34)：Cytogenetic Abnormality t(3;5)(q25;q34) present; t(6;11)(q27;q23)：Cytogenetic Abnormality t(6;11)(q27;q23) present; t(9;11)(p22;q23)：Cytogenetic Abnormality t(9;11)(p22;q23) present; t(10;11)(p11.2;q23):Cytogenetic Abnormality t(10;11)(p11.2;q23) present; t(11:19)(q23:p13.1): Cytogenetic Abnormality t(11:19)(q23:p13.1) present; inv(16):Cytogenetic Abnormality Chromosomal Inversion Chromosome 16 present; del7q:Cytogenetic Abnormality Deletion Mutation 7q present; del9q:Cytogenetic Abnormality Deletion Mutation 9q present;monosomy5*:Cytogenetic Abnormality Monosomy 5 present; monosomy7*:Cytogenetic Abnormality Monosomy 7 present;trisomy8: Cytogenetic Abnormality Trisomy Chromosome 8 present ;trisomy21: Cytogenetic Abnormality Trisomy Chromosome 21 present ;MLL: Cytogenetic Abnormality Translocations Involving MLL1 (KMT2A) Gene present; MinusY: Cytogenetic Abnormality Monosomy Chromosome Y Present; MinusX: Cytogenetic Abnormality Monosomy Chromosome X Present; Cytogenetic Complexity: Cytogenetic Abnormality Number of abnormalities;Primary Cytogenetic Code：Cytogenetic Abnormality Predominant Classification Type；FLT3_ITD: FLT3 Internal Tandem Duplication present; NPM：mutation of the NPM gene present; CEBPA: mutation of the CEBPA gene present; WT1: mutation of the WT1 gene present; FLT3_PM: FLT3 point mutation at codon 835-836 present; CKIT_EXON8: mutation of the exon 8 on KIT gene present; CKIT_EXON17: mutation of the exon 17 on c-kit gene present; MRD1:Minimal Residual Disease At End First Course; CR1: The remission status at the end of the first course of therapy determined by morphologic evaluation of marrow; <5% blast = CR; COG: Children's Oncology Group; Protocol: Children's Oncology Group Clinical Study Protocol; HSCT: Stem Cell Transplant During First Complete Remission; ELN: European LeukemiaNet; NUP98 fusion, KMT2A-r, RUNX1-RUNX1T1, CBFB-MYH11: Gene Fusion by RNA or

Whole Genome Sequencing or Karyotyping Identification.

Tables S2. Gene and coefficient list of P-AML-5G and two LSC models established for adult or pediatric AML

**P-AML-5G**	
COL23A1(ENSG00000050767,coeff=0.00024149)，TTC38(ENSG00000075234,coeff=0.00029096)，RNFT1(ENSG00000189050,coeff=0.00054436)，ZNF775(ENSG00000196456,coeff=-0.000674)，CRNDE(ENSG00000245694,coeff=0.0001792)	
**LSC17 model**	**REF: Docking, T Roderick et al. "A clinical transcriptome approach to patient stratification and therapy selection in acute myeloid leukemia." Nature communications vol.**
MMRN1(ENSG00000138722,coeff=0.0258)，DPYSL3(ENSG00000113657,coeff=0.0284)，CDK6(ENSG00000105810,coeff=-0.0704)，LAPTM4B(ENSG00000104341,coeff=0.00582)，AKR1C3(ENSG00000196139,coeff=-0.0402)，ARHGAP22(ENSG00000128805,coeff=-0.0138)，EMP1(ENSG00000134531,coeff=0.0146)，SOCS2(ENSG00000120833,coeff=0.0271)，NYNRIN(ENSG00000205978,coeff=0.00865)，KIAA0125(ENSG00000226777,coeff=0.0196)，GPR56(ENSG00000205336,coeff=0.0501)，CD34(ENSG00000174059,coeff=0.0338)，SMIM24(ENSG00000095932,coeff=-0.0226)，CPXM1(ENSG00000088882,coeff=-0.0258)，DNMT3B(ENSG00000088305,coeff=0.0874)，ZBTB46(ENSG00000130584,coeff=-0.0347)，NGFRAP1(ENSG00000166681,coeff=0.0465)	
**LSC6 model**	**Elsayed, A. H., Rafiee, R., Cao, X., Raimondi, S., Downing, J. R., Ribeiro, R., Fan, Y., Gruber, T. A., Baker, S., Klco, J., Rubnitz, J. E., Pounds, S., & Lamba, J. K. (2020). A six-gene leukemic stem cell score identifies high risk pediatric acute myeloid leukemia. Leukemia, 34(3), 735–745.**
SPINK2(ENSG00000128040,coeff=0.109)，SOCS2(ENSG00000120833,coeff=0.141)，FAM30A(ENSG00000226777,coeff=0.0516)，GPR56(ENSG00000205336,coeff=0.054)，CD34(ENSG00000174059,coeff=0.0171)，DNMT3B(ENSG00000088305,coeff=0.189)	

Note: LSCC, leukemic stem cell.

Tables S3. Definition of risk classification systems for P-AML (COG and expanded-COG-AAML1831) and adult AML (2022ELN)

**COG**	REF: Gemtuzumab ozogamicin in children and adolescents with de novo acute myeloid leukemia improves event-free survival by reducing relapse risk: results from the randomized phase III Children's Oncology Group trial AAML0531. *J Clin Oncol*. 2014;32(27):3021-3032<br>REF: Getz KD, Sung L, Ky B, Gerbing RB, Leger KJ, Leahy AB, Sack L, Woods WG, Alonzo T, Gamis A, Aplenc R. Occurrence of Treatment-Related Cardiotoxicity and Its Impact on Outcomes Among Children Treated in the AAML0531 Clinical Trial: A Report From the Children's Oncology Group. J Clin Oncol. 2019 Jan 1;37(1):12-21.
favorable	the presence of t(8;21)(q22;q22), inv(16)(p13.1q22), or t(16;16)(p13.1;q22)
adverse	presence of monosomy 7, monosomy 5/5q deletion, or persistent disease (PD) *at the end of first course of induction therapy, or with FLT-3 internal tandem duplication high allelic ratio (> 0.4; FLT3-ITD HAR)
Notes	Cytogenetics outweighed PD, whereas FLT3-ITD HAR outweighed favorable cytogenetics.
**expanded-COG-AAML1831**	REF1: Kim H. Treatments for children and adolescents with AML. *Blood Res*. 2020;55(S1):S5-S13. doi:10.5045/br.2020.S002<br>REF2: Adam J. Lamble, Rhonda E. Ries, Todd A. Alonzo, Yi-Cheng Wang, Jason E Farrar, Benjamin J. Huang, Matthew A. Kutny, Jessica A. Pollard, Richard Aplenc, Alan S. Gamis, Edward A. Kolb, Todd M. Cooper, Soheil Meshinchi; Expanding the High-Risk Definition for Children with Newly Diagnosed Acute Myeloid Leukemia. *Blood* 2022; 140 (Supplement 1): 3393–3394.
favorable	(8;21)(q22;q22) RUNX1-RUNX1T1<br>nv(16)/t(16;16)(p13.1q22) CBFB-MYH11<br>NPM1 mutations<br>Biallelic CEBPA mutation
adverse	inv(3)(q21q26.3)–MECOM-RPN1 fusion<br>t(6;9)(p23;q34.1)(DEKDEK-NUP214)<br>Monosomy 7<br>Monosomy 5/5q<br>Monosomy 5/5q-[EGR1(5q31)deleted]<br>KMT2A(MLL)(11q23.3)<br>-t(4;11)(q21;q23)<br>-t(6;11)(q27;q23)<br>-t(10;11)(p11.2;q23)<br>-t(10;11)(p12;q23)<br>-t(11;19)(q23;p13.3)<br>NUP98(11p15.5)<br>12p: Rearrangement or loss of ETV6<br>t(16;21)(p11;q22)(FUS-ERG)<br>FLT3/ITD+with allelic ratio>0.1%<br>CBFA2T3-GLIS2<br>RAM phenotype<br>KAT6A (8p11.21) Fusion (10p12)(for patients who are 90 days or older)<br>Non-KMT2A-MLLT10 Fusions<br>CEBPA co-occurring with CSF3R (included by REF2)<br>CREBBP mutations (included by REF2)<br>TP53 mutations (included by REF2)<br>IDH1/2 mutations in the absence of NPM1 (included by REF2)<br>fusions involving the ETS family （ERG, ETV1, ETV4 and ETV5) (included by REF2)<br>KMT2A-partial tandem duplications(included by REF2)
**2022ELN**	REF: Döhner H, Wei AH, Appelbaum FR, et al. Diagnosis and management of AML in adults: 2022 recommendations from an international expert panel on behalf of the ELN. Blood. 2022;140(12):1345-1377.<br>REF: Lachowiez, Curtis A et al. "Comparison and validation of the 2022 European

	LeukemiaNet guidelines in acute myeloid leukemia." Blood advances, bloodadvances.2022009010. 28 Nov. 2022
Favorable	• t(8;21)(q22;q22.1)/*RUNX1*::*RUNX1T1*†,‡<br>• inv(16)(p13.1q22) or t(16;16)(p13.1;q22)/ *CBFB*::*MYH11*†,‡<br>• Mutated *NPM1*†,§ without *FLT3*-ITD<br>• bZIP in-frame mutated *CEBPA*//
Intermediate	• Mutated *NPM1*†,§ with *FLT3*-ITD<br>• Wild-type *NPM1* with *FLT3*-ITD (without adverse-risk genetic lesions)<br>• t(9;11)(p21.3;q23.3)/*MLLT3*::*KMT2A*†,¶<br>• Cytogenetic and/or molecular abnormalities not classified as favorable or adverse
Adverse	• t(6;9)(p23.3;q34.1)/*DEK*::*NUP214*<br>• t(v;11q23.3)/*KMT2A*-rearranged#<br>• t(9;22)(q34.1;q11.2)/*BCR*::*ABL1*<br>• t(8;16)(p11.2;p13.3)/*KAT6A*::*CREBBP*<br>• inv(3)(q21.3q26.2) or t(3;3)(q21.3;q26.2)/ *GATA2*, *MECOM(EVI1)*<br>• t(3q26.2;v)/*MECOM*(*EVI1*)-rearranged<br>• −5 or del(5q); −7; −17/abn(17p) !<br>• Complex karyotype,** monosomal karyotype††<br>• Mutated *ASXL1, BCOR, EZH2, RUNX1, SF3B1, SRSF2, STAG2, U2AF1, and/or ZRSR2*‡‡<br>• Mutated *TP53*[a]
Notes	§ AML with NPM1 mutation and adverse-risk cytogenetic abnormalities are categorized as adverse-risk.<br>// Only in-frame mutations affecting the basic leucine zipper (bZIP) region of CEBPA, irrespective whether they occur as monoallelic or biallelic mutations, have been associated with favorable outcome.<br>¶The presence of t(9;11)(p21.3;q23.3) takes precedence over rare, concurrent adverse-risk gene mutations.<br>#Excluding KMT2A partial tandem duplication (PTD).<br>!Abn(17p) were defined as loss of 17p13 (TP53 locus) such as monosomy 17, deletion (17p), isochromosome 17q (i(17q)), addition (17p) or other abnormalities that disrupt the 17p13 locus.<br>**Complex karyotype: ⩾3 unrelated chromosome abnormalities in the absence of other class-defining recurring genetic abnormalities; excludes hyperdiploid karyotypes with three or more trisomies (or polysomies) without structural abnormalities.<br>††Monosomal karyotype: presence of two or more distinct monosomies (excluding loss of X or Y), or one single autosomal monosomy in combination with at least one structural chromosome abnormality (excluding core-binding factor AML).<br>‡‡ For the time being, these markers should not be used as an adverse prognostic marker if they co-occur with favorable-risk AML subtypes.<br>aTP53 mutation at a variant allele fraction of at least 10%, irrespective of the TP53 allelic status (mono- or biallelic mutation); TP53 mutations are significantly associated with AML with complex and monosomal karyotype.

Notes: ELN: European LeukemiaNet; COG: Children's
